# A scanning dynamic collimator for spot-scanning proton minibeam production

**DOI:** 10.1038/s41598-021-97941-w

**Published:** 2021-09-15

**Authors:** Marios Sotiropoulos, Yolanda Prezado

**Affiliations:** Institut Curie, Université PSL, CNRS UMR3347, Inserm U1021, Signalisation Radiobiologie et Cancer, 91400 Orsay, France

**Keywords:** Radiotherapy, Physics

## Abstract

In proton minibeam radiation therapy, proton minibeams are typically produced by modulating a uniform field using a multislit collimator. Multislit collimators produce minibeams of fixed length and width, and a new collimator has to be manufactured each time a new minibeam array is required, limiting its flexibility. In this work, we propose a scanning dynamic collimator for the generation of proton minibeams arrays. The new collimator system proposed is able to produce any minibeam required on an on-line basis by modulating the pencil beam spots of modern proton therapy machines, rather than a uniform field. The new collimator is evaluated through Monte Carlo simulations and the produced proton minibeams are compared with that of a multislit collimator. Furthermore, a proof of concept experiment is conducted to demonstrate the feasibility of producing a minibeam array by repositioning (i.e. scanning) a collimator. It is concluded that besides the technical challenges, the new collimator design is producing equivalent minibeam arrays to the multislit collimator, whilst is flexible to produce any minibeam array desired.

## Introduction

Proton Minibeam radiation therapy (pMBRT) is a novel radiotherapy approach based on a strong dose modulation^1,2^. pMBRT irradiations use typically planar minibeams 0.5 to 1 mm width spaced by 2 to 4 mm^3,4^.

In this non-homogeneous dose deposition pattern, regions of low and high dose are observed^4^. Contrary to conventional proton therapy, pMBRT uses multiple coulomb scatting to its advantage: proton minibeams get increasingly wider as a function of depth, which may result in a homogeneous target dose coverage^1^, while normal tissues at the entrance will benefit from the spatial fractionation of the dose. Indeed, pMBRT has already demonstrated a significant reduction of normal tissue toxicities both in the skin^5,6^ and brain^6,7^. Additionally, an equivalent or superior life span has been observed in tumour-bearing rats treated with pMBRT as compared to standard (broad beam) proton therapy (PT)^8–10^.

One of the crucial issues in pMBRT is its technical implementation, and, particularly, the generation of minibeams. The generation method influences the shape and size of minibeams, the peak-to-valley dose ratio (PVDR) but as well potential neutron contamination^11,12^. All of these aspects have an impact on the biology response^4^.

Up to now, all MBRT experiments with protons of clinically relevant energies (i.e. ≥ 70 MeV) have been performed with planar minibeams generated using multislit collimators^4^. Those were placed either at the end of a passive beamline^13^ or at the exit of a pencil beam scanning nozzle^14^. The main advantage of minibeam generation with multislit collimators is that it enables its implementation at any proton therapy centre^15^. The main drawbacks are their inefficiency, inflexibility (a custom collimator may have to be fabricated for each case) and introduce a source of secondary neutrons close to the patient^12,16^.

Multi-leaf collimators (MLCs) and dynamic collimators (DCs) have been implemented in active scanning proton beam therapy for penumbra reduction^17,18^. Potentially this technology could be used to generate minibeams. However, there are several limitations that inhibit direct translation in pMBRT: (i) achieving the positioning accuracy needed for minibeams would be extremely difficult as the leafs/blocks should be moved with an accuracy of a few micrometer, (ii) following the beam divergence would be very challenging, and (iii) more likely the device would be too large to allow for small air gaps.

A potential solution may be the use of magnetic focusing^19^. So far, the only facility having implemented magnetic focusing for pMBRT is the SNAKE microprobe in Munich^20^. However, the maximum beam energy at this installation is limited to 20 MeV^5^ which is too low for most clinical applications. Schneider et al. have proposed a new nozzle^19,21^. Despite the advantages of this minibeam generation method, the fact of involving a new/different nozzle, make it difficult to be retrofitted in already existing facilities and most likely, it could only be coupled to newly constructed beamlines.

Here, the concept of a scanning dynamic collimator system for proton minibeam generation is proposed. It aims to overcome the limitations of the multislit collimator system providing the flexibility desired for the investigation of the proton minibeam properties, both at the physical and biological layer. In this system a dynamic collimator that allows the selection of the minibeam size is coupled with a scanning capable system that gives the ability to generate any minibeam array required. Firstly, we present the system and evaluate through Monte Carlo simulations its ability to create minibeam arrays similar to a multislit collimator. Then a proof of concept experiment is conducted in order to evaluate the feasibility of the scanning collimator minibeam generation method when combined with a proton spot scanning system.

## Materials and methods

### A proton minibeam scanning dynamic collimator

The concept of a scanning dynamic collimator for proton minibeam generation is proposed. This scanning dynamic collimator is adapted to the pencil beam scanning capabilities of the modern proton therapy machines and allows to select slit width and length, and centre-to-centre distance dynamically in order to produce a fully customized minibeam array.

**Figure 1:**
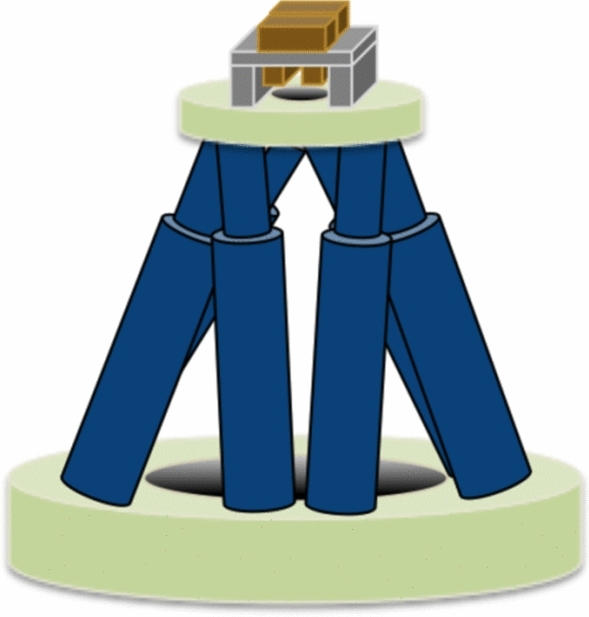
Conceptual sketch of the proton minibeam scanning dynamic collimator. The two-plane dynamic collimator is on top of a hexapod.

The system consists of two main elements (see Fig. 1): (i) a single slit dynamic collimator that allows the selection of the desired single slit parameters (i.e. width and length) and (ii) the scanning system that allows repositioning the dynamic collimator to form the minibeam array. For the scanning system, a hexapod was selected. In addition, the rotational degrees of freedom inherent to the hexapod allow rotating the single slit collimator in order to follow the beam divergence. Furthermore, the hexapod gives the additional capability of fine trimming to achieve a good alignment of the system.

#### Dynamic collimator designs

Two dynamic collimator designs are proposed: (i) an aperture-like collimator and (ii) a two-plane collimator. Another option, which however limits the flexibility of the system, would be an interchangeable (fixed) single slit collimator. In this case, a set of predefined slit widths and length would be available.

The collimator blocks are made of brass, since brass is the material of choice for proton minibeam collimators^11,13,14^. This choice is based on a compromise between a good collimation that leads to a high entrance peak-to-valley dose ratio and neutron production^12^. In this investigation the collimators used have a thickness of 5 cm and the collimators lateral extension is 5 cm, unless otherwise stated.

##### Aperture collimator

**Figure 2:**
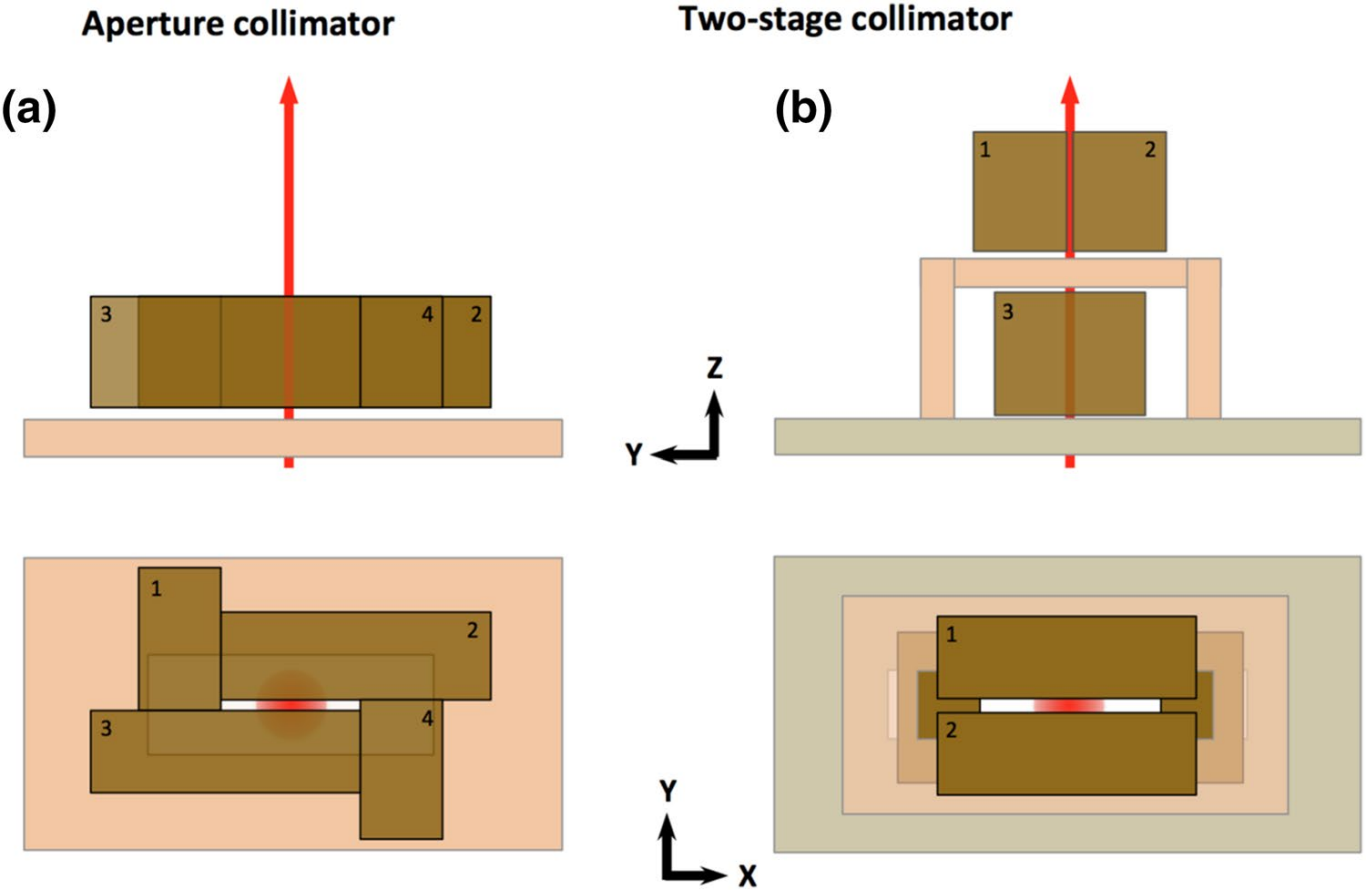
Dynamic collimator designs: (**a**) aperture collimator and (**b**) two-plane collimator. The proton beam direction is depicted with red.

This collimator consists of 4 blocks of radiation blocking material (Fig. 2a). Each block has two degrees of freedom. To change the slit length (x direction) collimator 1 and 2 moves on the opposite direction to collimator 3 and 4. To change the slit width, collimator 1 and 3 moves on the opposite direction to collimator 2 and 4.

The realization of this dynamic collimator has many technical challenges. The blocks have to move with high accuracy to limit the gap in between. A tongue and groove approach, similar to that used in multi-leaf collimators could be included. This collimator results in a small footprint and would be more likely suited for small apertures.

##### Two level collimator

In this dynamic collimator design the length-wise and width-wise collimation is done at different planes (Fig. 2b). The top collimator (i.e. the one closer to the target) controls the slit width while the other limits the slit length. Overall, is a much simpler design compared to the aperture collimator, allows for making a collimator with increased length and can result to a more conical design suitable to reach close to the patient.

#### Scanning collimator

The scanning capabilities of the proposed system are exploited in order to create the minibeam array. Two ways of scanning are proposed, the point-by-point and the line-by-line scanning collimator.

##### Point-by-point scanning collimator

**Figure 3:**
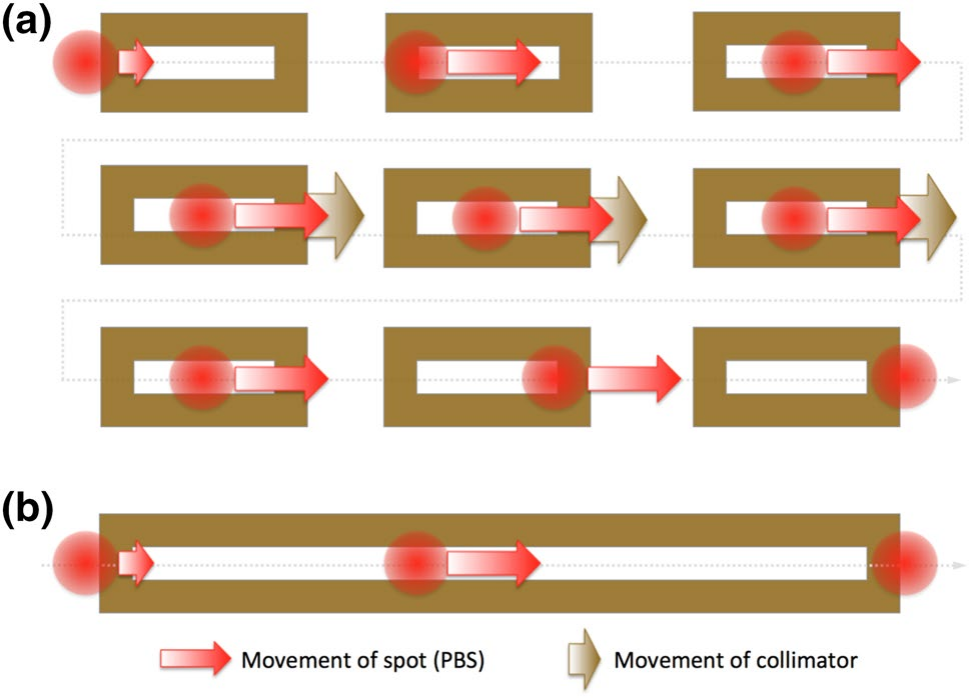
Demonstration of spot scanning collimation: (**a**) point-by-point scanning collimator. The beam spot starts outside the collimator, enters the collimator and when is at the centre of the collimator, the collimator starts moving. When the collimator is at its final position, the beam exits the collimator. (**b**) Line-by-line scanning collimator. The collimator has the size of the required minibeam length and remains still during the scanning.

In the point-by-point scanning collimator system the length of the slit can be fixed in such length that one pencil beam spot in about the centre of the slit passes with minimal collimation at the slit length direction (see “Design parameters affecting the dose distributions”). In order to create a minibeam, the single slit collimator moves with the pencil beam spot. As illustrated in Fig. 3a, initially the collimator is static. The beam moves until it reaches the centre of the collimator. When the beam is at the centre of the collimator, the collimator starts its movement, following the beam. When the collimator reaches its final position, the collimator stops and allows the beam to finish the scanning. Afterwards, the collimator is positioned for the next minibeam in the minibeam array, to compile the full array.

This scanning approach could benefit from an aperture like collimator (see “Aperture collimator”).

##### Line-by-line scanning collimator

Another possibility is the maximum length of the single slit dynamic collimator to be large enough to produce the maximum desired minibeam. The two-level collimator, which is more suitable for large field lengths, would be more appropriate. For each desired minibeam, the collimator takes the appropriate length; the spot is scanned over the collimator (see Fig. 3b) producing the minibeam. Then the collimator is positioned for the next minibeam.

### Monte Carlo evaluation of the scanning collimator system

The proposed system was evaluated by means of Monte Carlo simulations. The nozzle of the ICPO beam line was modelled using the TOPAS simulation tool^22,23^, as has been reported before^14^. The beam parameters from the characterisation from De Marzi et al^24^ were used. In this parameterization the beam spot at the vacuum window is characterized by the (i) energy and energy spread of the beam, (ii) spot size, (iii) beam divergence and (iv) beam correlation. Using this parameterization, the magnetic field strength of the two scanning magnets can be calculated as a function of the spot position at the isocentre, allowing the creation of any field needed.

#### Design parameters affecting the dose distributions

The advantage of the hexapod system is its capability to follow the beam divergence. When a position other than the central (i.e. without applying a magnetic field at the scanning magnet) is required, the beam is deflected to reach this position. The importance of following this deflection angle is demonstrated. Two cases are investigated: (i) the case of repositioning and rotating the collimator following the beam deflection and (ii) repositioning the collimator so that the beam spot targets the collimator slit, but without rotating the collimator (see supplementary material, Sect. 2.2).

In the case of the point-by-point scanning collimator the minimum size of the collimator that allows an undisturbed beam on the slit length direction is evaluated. To identify the appropriate slit length that creates an undisturbed beam, different slit lengths (l = 1, 2, 3, 5, and 7 cm) of a fixed collimator are compared with a parallel plate collimator. A slit width of 400 μm is used.

The performance in terms of dose distributions of the two-plane collimator is compared to that of a single slit collimator. The aperture collimator is not investigated as is considered to be equivalent to the single slit collimator. The slit width is 400 μm with a 5 cm slit length.

#### Scanning proton minibeam generation

To evaluate the new scanning collimator design, the minibeams generated by a multislit collimator were compared with the scanning single slit and two-plane collimator. To produce the minibeam array with the scanning collimators, the collimators and beam were repositioned accordingly.

### Proof of concept experiment

In the previous section a scanning dynamic collimator for proton minibeam production was proposed. In this section we conduct a proof of concept experiment in order to evaluate the feasibility of the scanning component of the minibeam collimator. In this proof of concept experiment we focus on generating a minibeam array by repositioning the collimator. Therefore, rather than having a dynamic collimator, a single slit collimator is used. The single slit is repositioned to produce the desired minibeam array. This allows to confirm that the new collimator design would work when positional uncertainties are present; i.e. when combined with a real proton therapy beamline.

#### Experimental setup

**Figure 4:**
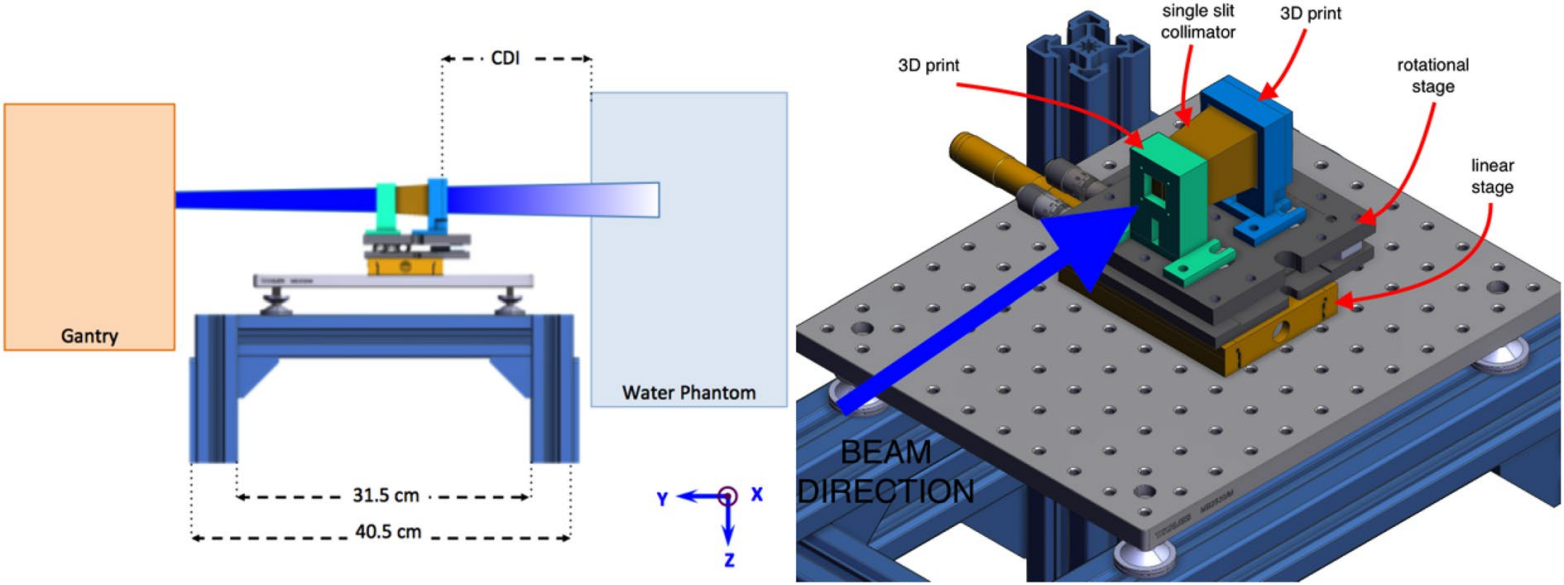
Setup of the proof of concept experiment. A single slit collimator is placed on top of a translational and rotational stage. The left surface of the water phantom is at the isocentre. The collimator to isocentre distance (CDI) is 10 cm.

The experimental setup consists of the stand with the single slit collimator placed between the gantry and the water tank (Fig. 4; left). The tank was empty and was placed as a stable surface where the measurement films could be placed. The collimator is placed on top of a linear (M-UMR8.51 with the BM17.51 µm head from Newport, Irvine, California, USA) and rotational stage (PY004/M from ThorLabs, Newton, New Jersey, USA) allowing 2 degrees of freedom (Fig. 4; right). The collimator, linear and rotational stage system is located on an aluminium breadboard with 3 legs that allows to fine adjust the horizontal alignment of the manual stages-collimator assembly. The collimator slit has a length of 2.5 cm and a width of 400 μm and was positioned at a vertical orientation, at a collimator to isocentre distance (CDI) of 10 cm.

**Figure 5:**
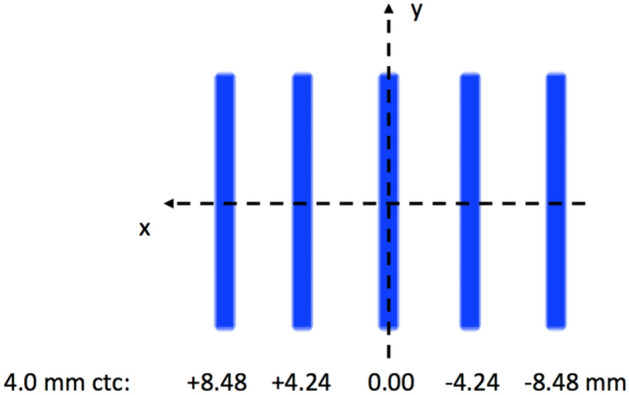
Sketch of the expected minibeam pattern at the isocentre (i.e. film position). 5 minibeams are demonstrated.

The un-modulated proton field was 3 spots wide horizontally (x-axis) and 8 spots vertically (y-axis). The field was repositioned so that it remained centred to the slit, when the slit had to be moved to a new position in order to form another minibeam. The horizontal displacement and angle of the collimator were calculated for each slit (see supplementary material, Sect. 3.2), for a collimator with a centre-to-centre distance of 4.0 mm. However, as some misalignment of the experimental apparatus might happen, these positions were used as reference (see “Measurements”, step 2 for more details). The projected minibeam pattern at the isocentre is shown on Fig. 5.

The minibeam array was measured with calibrated EBTXD (lot 08021701) radiochromic film on the surface of the (empty) water tank.

#### Measurements

The following procedure was followed for the experimental measurements:

1. The collimator system was initially aligned at the position of the central minibeam. That included some trial and error procedure where the position (divergence and displacement) of the collimator was changed and the minibeam pattern was inspected with a film. When the single minibeam pattern was satisfactory, the position of the slit was kept.
2. The slit was moved to the next position using the collimator position parameters that had been calculated. The corresponding proton spot pattern was used for the irradiation. Again, a trial and error procedure was used to evaluate the correct alignment. When a satisfactory single minibeam pattern was achieved, the position was kept and moved to the next position.
3. The previous process was repeated for all single slits in a minibeam array. When the necessary number of single minibeam arrays was reached, a full array was compiled by using the positions previously stored.

This process was followed for compiling a 3 and 5 minibeams array.

## Results

The concept of a scanning dynamic collimator was introduced in the previous section. In this section the design choices presented are evaluated and the minibeam dose distributions produced by the new design are compared with the dose distributions by the multislit collimator. Then the results from the proof of concept experiment are reported.

### Design parameters affecting the minibeam generation

The effect of the beam deflection angle, slit length and collimator design to the dose distributions was studied by means of Monte Carlo simulations.

**Figure 6:**
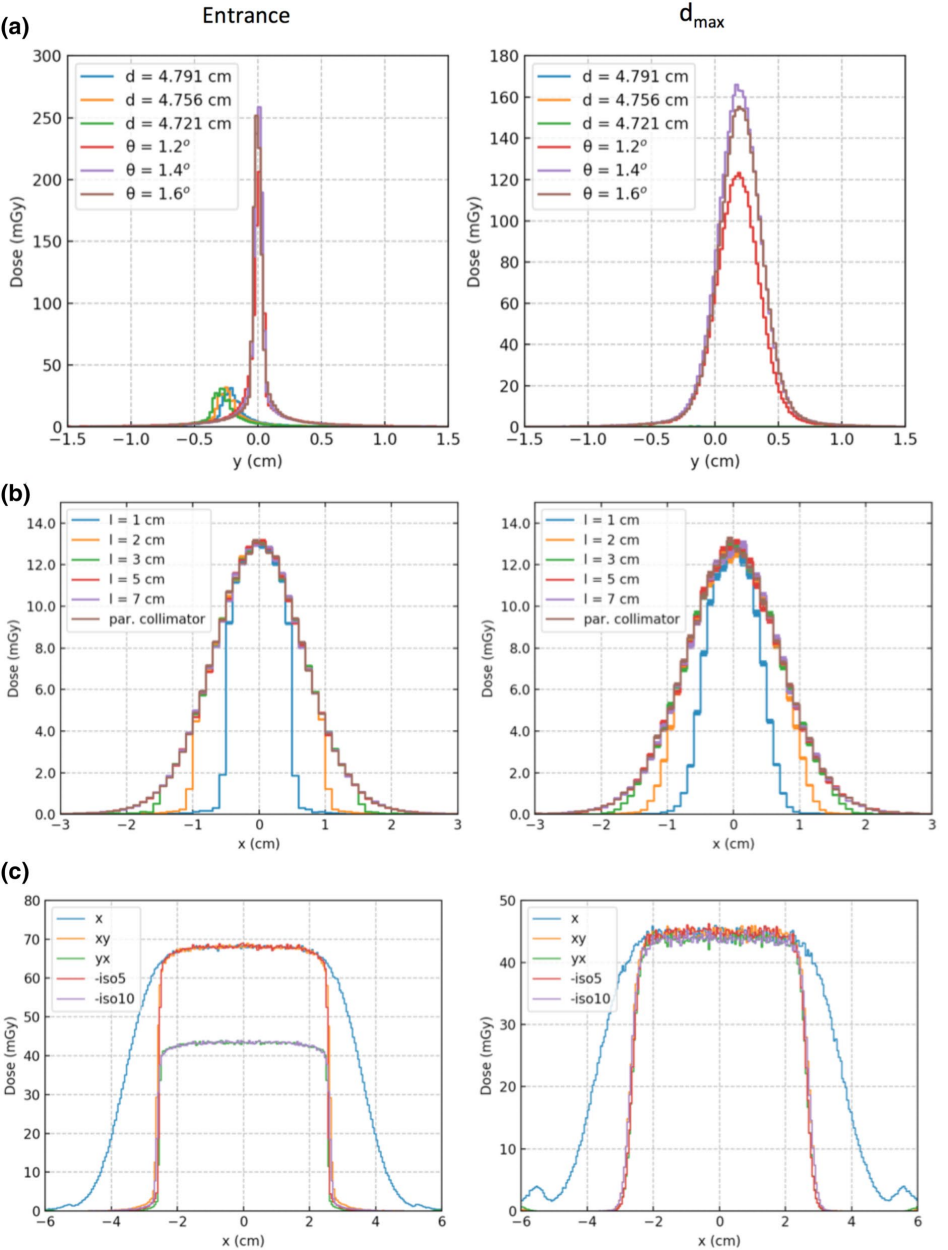
(**a**) Lengthwise profiles to evaluate the effect of following the beam divergence. Either the collimator is placed at different positions (d = 4.791, 4.756 and 4.721 cm) without following the deflection angle or rotated at different angle (θ = 1.2°, 1.4° and 1.6°), following the beam deflection angle. (**b**) Widthwise profiles produced by a single slit collimator with different width (l = 1, 2, 3, 5 and 7 cm) and a parallel collimator (i.e. l = ∞). (**c**) Dose profiles produced by different collimator designs: parallel collimator (‘x’), two-plane collimator with the lengthwise (‘xy’) or the widthwise (‘yx’) collimator placed closer to the phantom, and single slit collimator placed at 5 or 10 cm from the phantom (‘–iso5’ and ‘–iso10’ respectively). The profiles were calculated at the entrance (top row) and the position of the maximum dose (d_max_) for a 100 MeV proton beam.

Large reduction in the entrance dose was observed when the collimator was not rotated to follow the beam deflection angle, for a proton beam of 100 MeV (Fig. 6a). In the supplementary material, Sect. 2.2, the profiles for a beam of 150 MeV are also given. Also, when the collimator is not well aligned with the beam, reduction in the dose at the entrance and D_max_ was observed. These results demonstrate the necessity for following the beam deflection angle. In the proposed system this is achieved by the use of the hexapod.

In the case of the point-by-point scanning method a small collimator can be used. The minimum size of this collimator was calculated. In our proton system, for a beam of 100 MeV we get a spot of 6.7 mm at the isocentre (see supplementary material, Sect. 1.2). For this spot size, a slit length of 3 cm will allow the beam to get through the collimator with minimal loss (Fig. 6b). See also supplementary material Sect. 2.1 for results for a 150 MeV proton beam.

In the system proposed, the line-by-line scanning method will be significantly benefit by a two-plane collimator. The two-plane collimator is compared with the single slit collimator in terms of lateral profiles, for a proton beam of 100 MeV (Fig. 6c); see supplementary material, Sect. 2.3, for a beam of 150 MeV. The two-plane collimator is found equivalent to the single slit collimator. In the case of the two-plane collimator, the length wise collimator blocks should be placed close to the phantom surface, otherwise a significant reduction on the entrance dose is observed.

### Simulated minibeams

**Figure 7:**
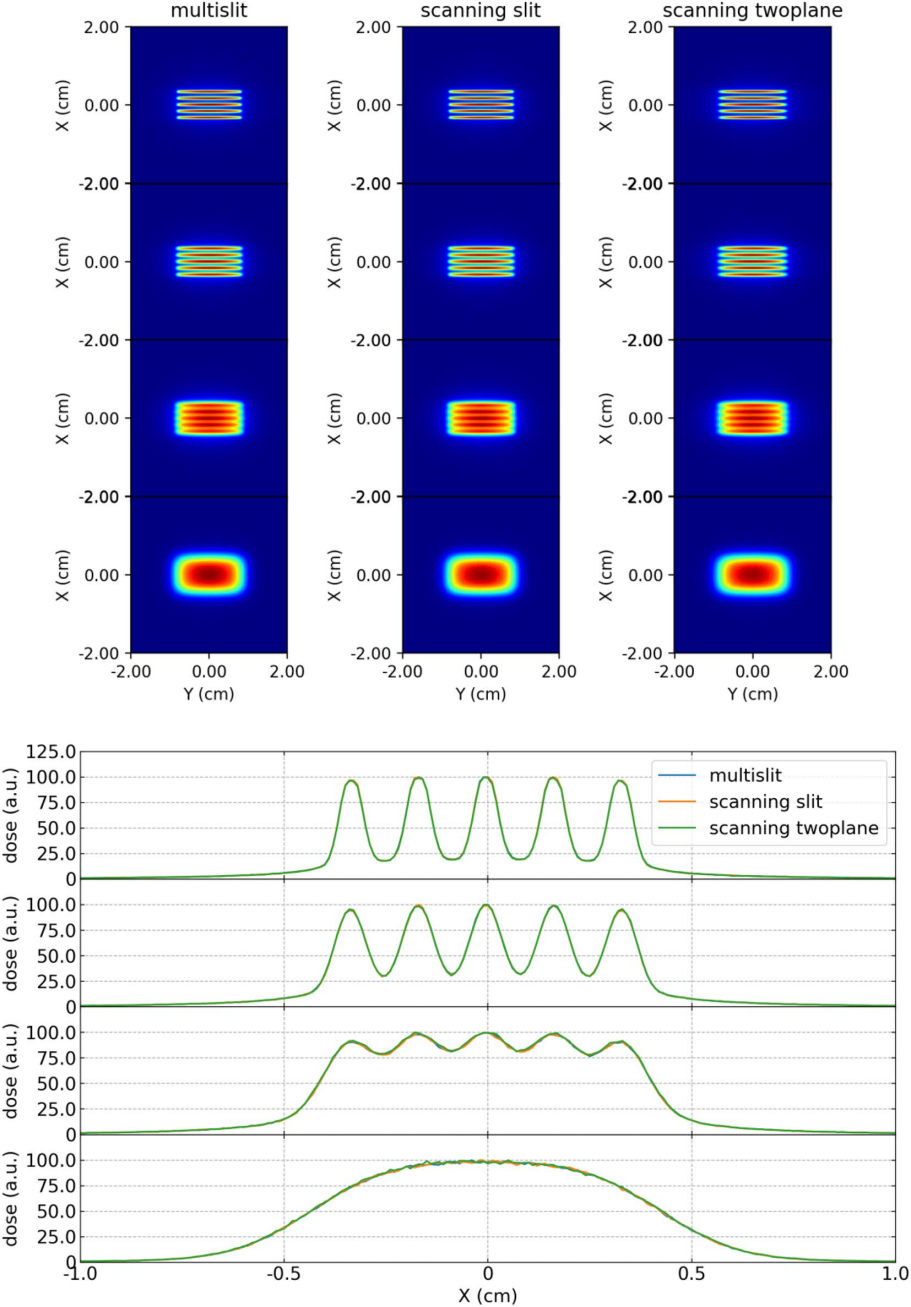
Minibeam dose distributions (top) and profiles (bottom) at the surface, 2 cm and 4 cm depth, and the depth of maximum dose, for the multislit, the scanning slit and the scanning two plane collimator.

The minibeams dose distributions produced by the multislit, scanning single slit and scanning two plane collimator are compared in Fig. 7, at the surface, 2 cm and 4 cm depth, and the depth of maximum dose. The produced minibeam profiles are equivalent for all depth, rendering the peak-to-valley dose ratio (PVDR) equal between all methods. The PVDRs at different depths for the multislit collimator are given in Table 1.

**Table 1:** Peak-to-valley dose ratio (PVDR) calculated for the multislit collimator at different depths. At the depth of the maximum dose the PVDR is not available as the minibeams are not anymore distinguishable but have been merged due to the multiple coulomb scattering.

Depth (cm)	PVDR
0	5.11 ± 0.06
2	3.29 ± 0.04
4	1.22 ± 0.01
D_max_ (7.63)	N/A

### Proof of concept experiment

**Figure 8:**
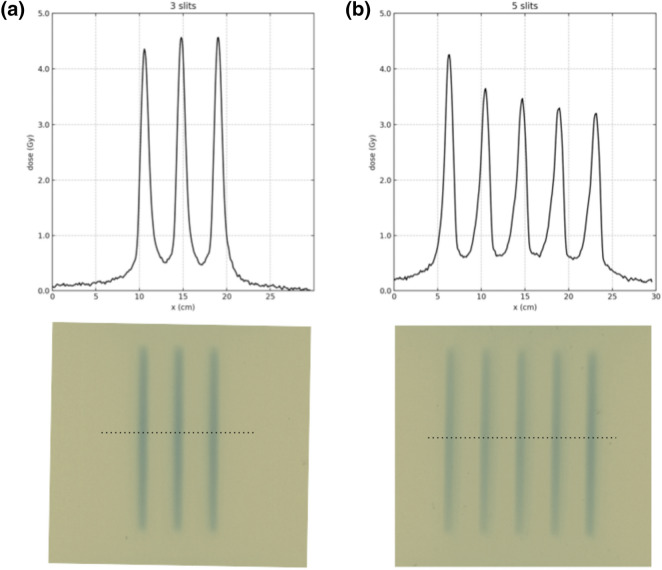
The films and corresponding dose profiles of the 3 (**a**) and 5 (**b**) slits minibeam array generated by the single slit scanning collimator.

Using the manual scanning collimator two minibeam arrays were created, one with 3 and one with 5 minibeams. The films demonstrating the 3 and 5 slits minibeam array generated by the scanning single slit collimator are shown in Fig. 8. The distances between the positions of the maximum dose (peaks) for each minibeam array was measured and are given in Table 2. A good agreement with the expected distance of 4.24 mm is observed.

**Table 2:** Distances between two adjacent minibeam peaks, for the 3 and 5 slit minibeam array.

Distance between peaks (mm)	
3 slits	5 slits
4.23	4.15
4.23	4.23
	4.23
	4.23

## Discussion

The concept of a scanning dynamic collimator for the generation of proton minibeams was presented for the first time. The system presented is based on a dynamic single slit collimator that is being repositioned by a hexapod to allow the creation of a minibeam array. To further confirm the feasibility of the design a proof of concept experiment were also conducted.

The new minibeam collimator concept proposed allows to dynamically select the characteristics of the minibeam array (slit width and length, and centre-to-centre distance of the minibeams). This results to a very flexible design that makes it possible to compile any desired minibeam array. The resulting device can be retrofitted to any machine and removed when is not needed. By using a hexapod for the scanning stage a conical shape can be achieved that will allow the collimator to be positioned as close to the patient as possible. Its weight is not expected to be more than 50 kg.

Alongside with the innovative aspect of the device come the technical challenges. Firstly, the scanning dynamic collimator needs to be synchronised with the beam delivery system, and remain at a good synchronization state during the irradiation. The synchronisation is significantly more challenging in the case of a point-by-point scanning method. However, the line-by-line scanning method can substantially reduce the necessity for online synchronization. The collimator could be moved to a new line at known intervals with the beam turned on and off accordingly. Secondly, the alignment (i.e. the position and angle of the collimator) of the system with regard to the beam spot positioning is very crucial. This can be further seen in the proof of concept experiment and in particular in the 5-slit minibeam array, where a misalignment is observed resulting to unequal peaks in the dose profile.

In the proof of concept experiment the combination of using manual stages for the movement and the film for the measurements had limited the alignment accuracy. The effect of the misalignment can be seen in particular in the 5 slit minibeam array (Fig. 8), where the intensity of the minibeam peaks is not constant. This is linked to a systematic error in the alignment. Further improvements in our proof of concept experimental method are envisaged, to improve the alignment. For example, a high-resolution screen detector such as the Lynx (IBA dosimetry, Schwarzenbruck, Germany) could be used to improve and accelerate the alignment procedure. We believe that a motorised system for the collimator positioning with a high-resolution detector system for the detection of the collimator correct position could solve the alignment problems.

## Conclusions

A new proton minibeam collimator was presented. The new collimator design introduces the concept of a scanning dynamic collimator for the production of a minibeam array. The new minibeam generation method is specifically adapted to modern pencil beam scanning proton therapy systems and allows to produce any minibeam length and width desired online.

Our simulations showed that the new collimator design is able to produce equivalent dose distributions with the multislit collimator that is currently in use. In addition, we conducted a proof of concept experiment to demonstrate the feasibility of the concept. In this experiment, a minibeam array was produced by repositioning a single slit collimator to pre-calculated positions.

## Supplementary Information

Supplementary Information.

## Data Availability

The datasets generated during and analyzed during the current study are available from the corresponding author on reasonable request.
