# Supplementary material for: A scanning dynamic collimator for spot-scanning proton minibeam production
Source: Sci Rep. 2021 Sep 15;11:18321. doi: 10.1038/s41598-021-97941-w (PMC8443660; doi:10.1038/s41598-021-97941-w)
Supplement: Supplementary file 1 — Supplementary Information. [file 41598_2021_97941_MOESM1_ESM.docx]

Supplementary material:

A scanning dynamic collimator for spot-scanning proton minibeam production

Marios Sotiropoulos^1^, Yolanda Prezado^1^

Institut Curie, Université PSL, CNRS UMR3347, Inserm U1021, Signalisation radiobiologie et cancer, 91400 Orsay, France

1. Proton beam spot characterization 1

1.1. Depth dose profiles 1

1.2. Spot size 2

2. Additional material 2

2.1. Single slit length evaluation 2

2.2. Importance of following the beam divergence 4

2.3. Comparing different collimator designs 6

3. Beam deflection angle 8

3.1. Scanning Magnet 1 (SM1) i.e. scanning along x-axis 8

3.1.1. E = 100 MeV 8

3.1.2. E = 150 MeV 11

3.2. Calculating the slit positions 14

# Proton beam spot characterization

The spots for a 100 and 150 MeV proton beam are characterized. The spots characteristics are measured on a water phantom with voxel size 0.5 mm x 0.5 mm x 0.5 mm positioned with its front surface at the isocentre.

## Depth dose profiles

At Figure 1‑1 the depth dose profiles are plotted and the D_max_ is calculated.


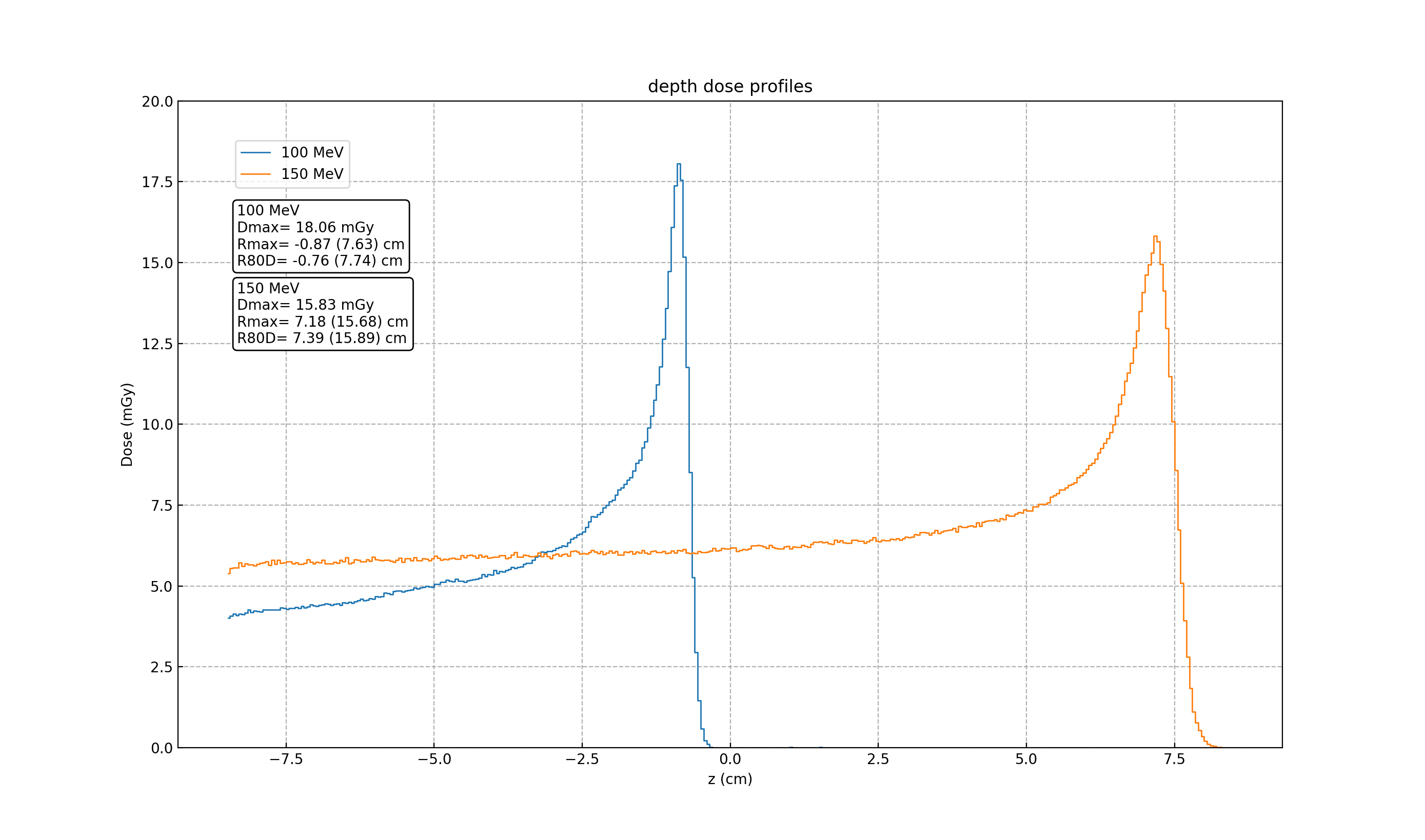


Figure 1‑1. Depth dose profile (DDP) and range estimation for the 100 and 150 MeV spots. In the parenthesis are the depth from the begging of the water phantom.

## Spot size

At Figure 1‑2 the spot size is measured at the entrance (that is the first ‘slice’ of the voxelized phantom) and at the depth of the maximum dose, D_max_.


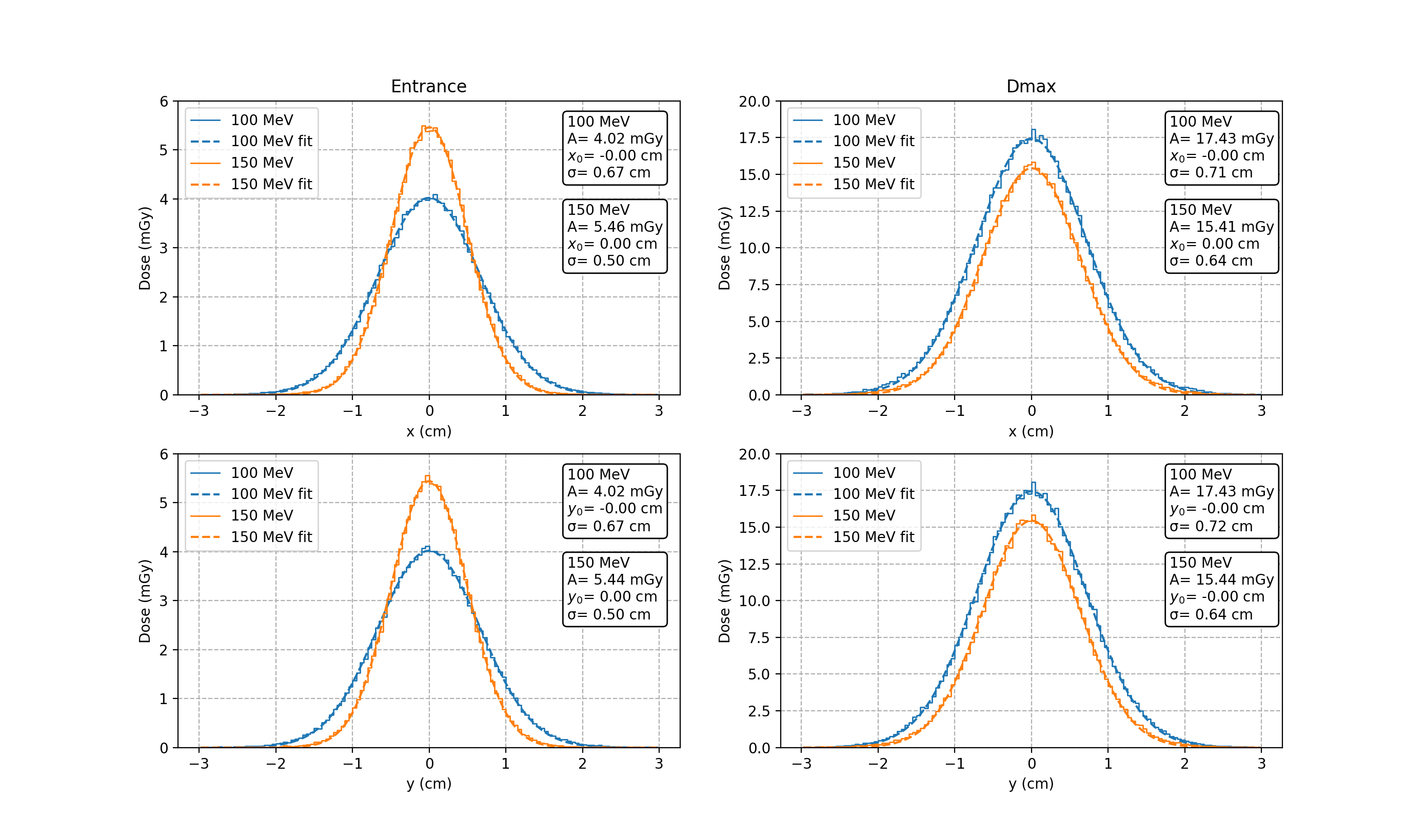


Figure 1‑2. Spot size dimensions for 100 and 150 MeV at the entrance and D_max_.

# Additional material

## Single slit length evaluation

In the case of the aperture collimator (or equally, in the case of a fixed slit collimator) for the point-by-point scanning method, the length of the collimator that allows an undisturbed beam on the slit length direction is calculated. A water tank with voxel size of 0.5 x 0.1 x 1 mm^3^ is placed with its front surface at the isocentre. A single spot of 50M protons are simulated.


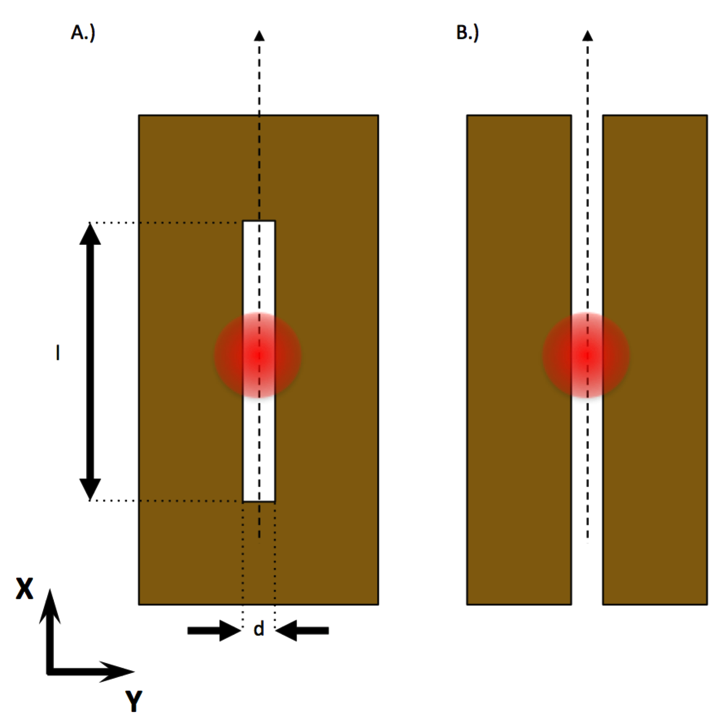


Figure 2‑1. A) Single slit collimator characterized from its length, l, and slit width, d. B.) Parallel plate collimator.

The length- and width-wise profiles for a slit with a length of 1, 2, 3, 5, 7 cm and a parallel collimator for a beam of 100 and 150 MeV are shown in Figure 2‑2 and Figure 2‑3 respectively.


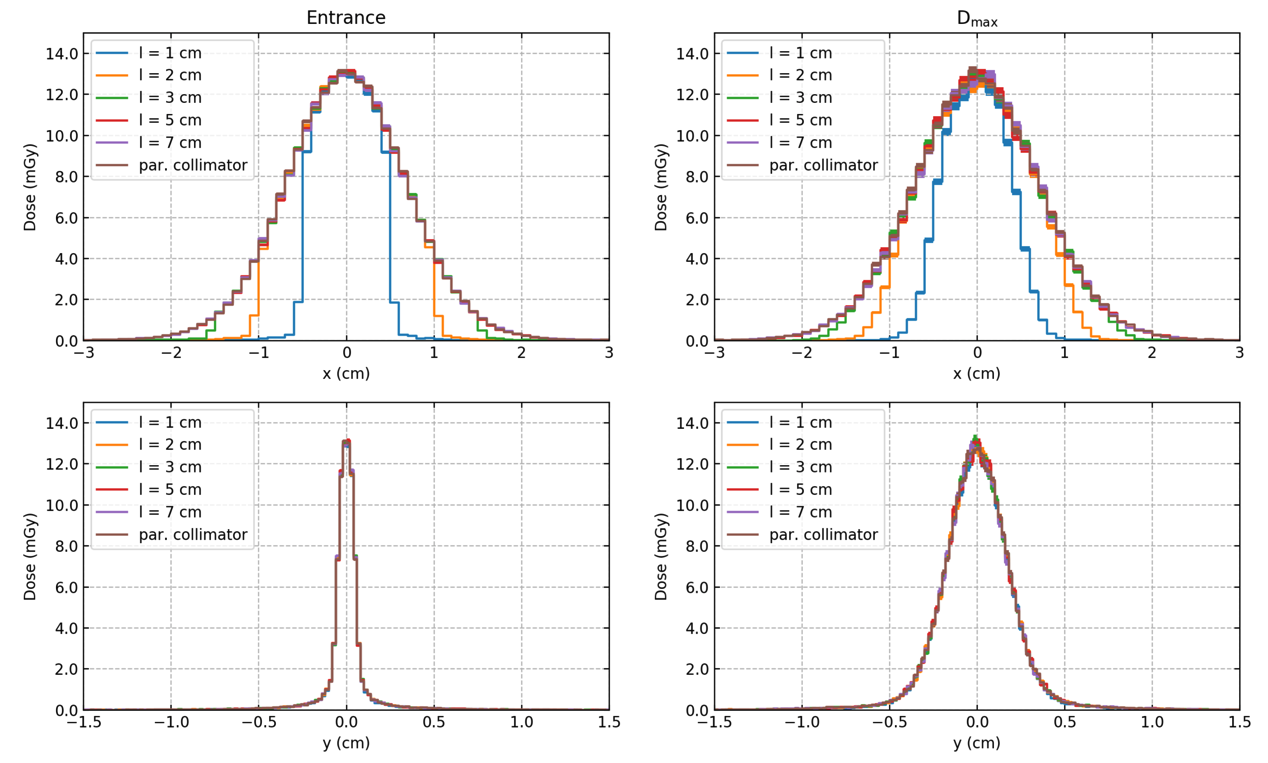


Figure 2‑2. Length- (top row) and width- (bottom row) wise profiles at the entrance and D_max_ for 100 MeV protons.


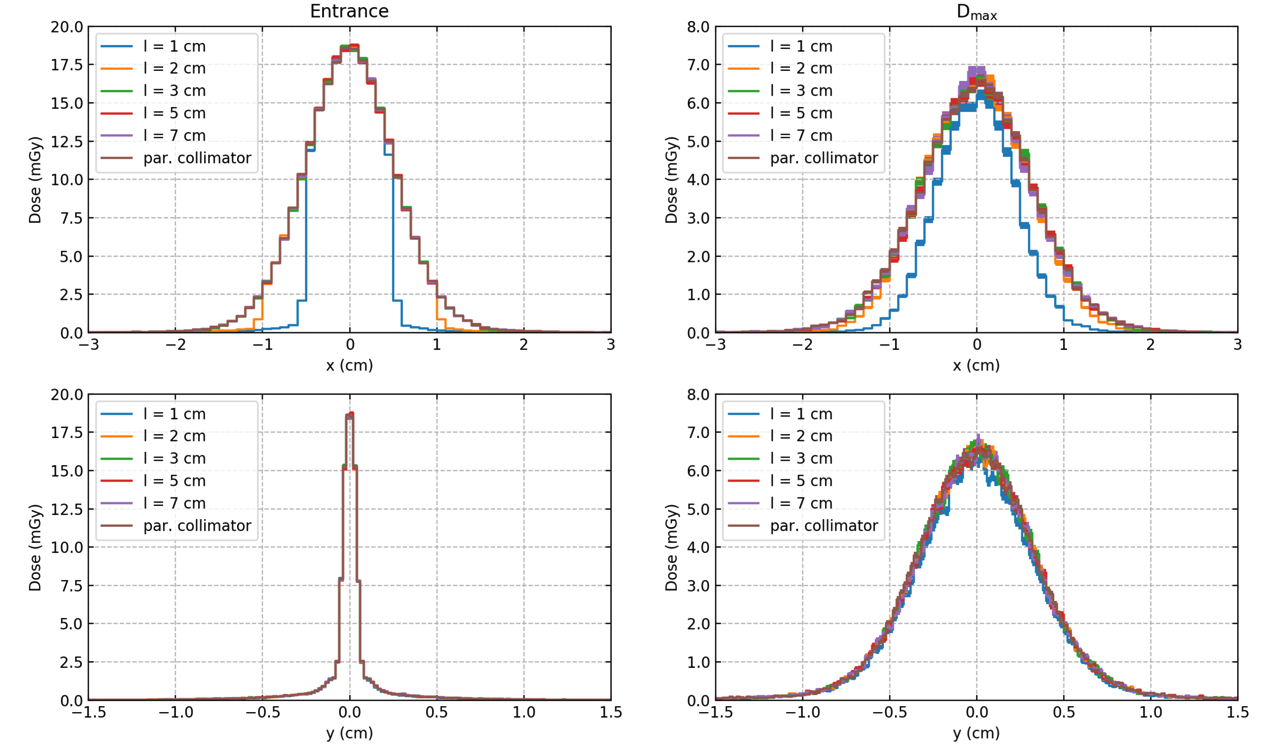


Figure 2‑3. Length- (top row) and width- (bottom row) wise profiles at the entrance and D_max_ for 150 MeV protons.

## Importance of following the beam divergence

As the beam spots scanned across the field they form a deflection angle, θ. The importance of rotating the collimator to follow the beam deflection angle is demonstrated in this section.

The simulation consists of the full ICPO beam line model and a voxelized water phantom (voxel size 0.5 x 0.1 x 1 mm^3^) with its front surface on the isocentre (Figure 2‑4). Two cases are investigated: case B, the case of repositioning and rotating the collimator following the beam deflection (Figure 2‑4B) and, case C, repositioning the collimator so that the beam spot targets the collimator slit, but without rotating the collimator (Figure 2‑4C).

Using simple trigonometry a relation between the displacement of the slit, the position of the spot at the isocentre and the deflection angle is found.

|  | $l_{1}=y_{1}-d\cdot tan\theta$ | Eq. 1 |
| --- | --- | --- |

The formula of equation 1 is used to calculate the position of the slit for a set of angles (1.2, 1.4 and 1.6 degrees), for each case. In case B, for each angle in the set the displacement (l1) is calculated to position the slit on the trajectory of the beam, as well as the collimator is rotated to follow the beam deflection angle. In case C only the collimator is displaced, without rotation. The position of the beam at the isocentre is y1 = 5 cm. A field consisting of a line of 17 spots separated by 3 mm at the isocentre is used, with 300M protons simulated per beam spot.


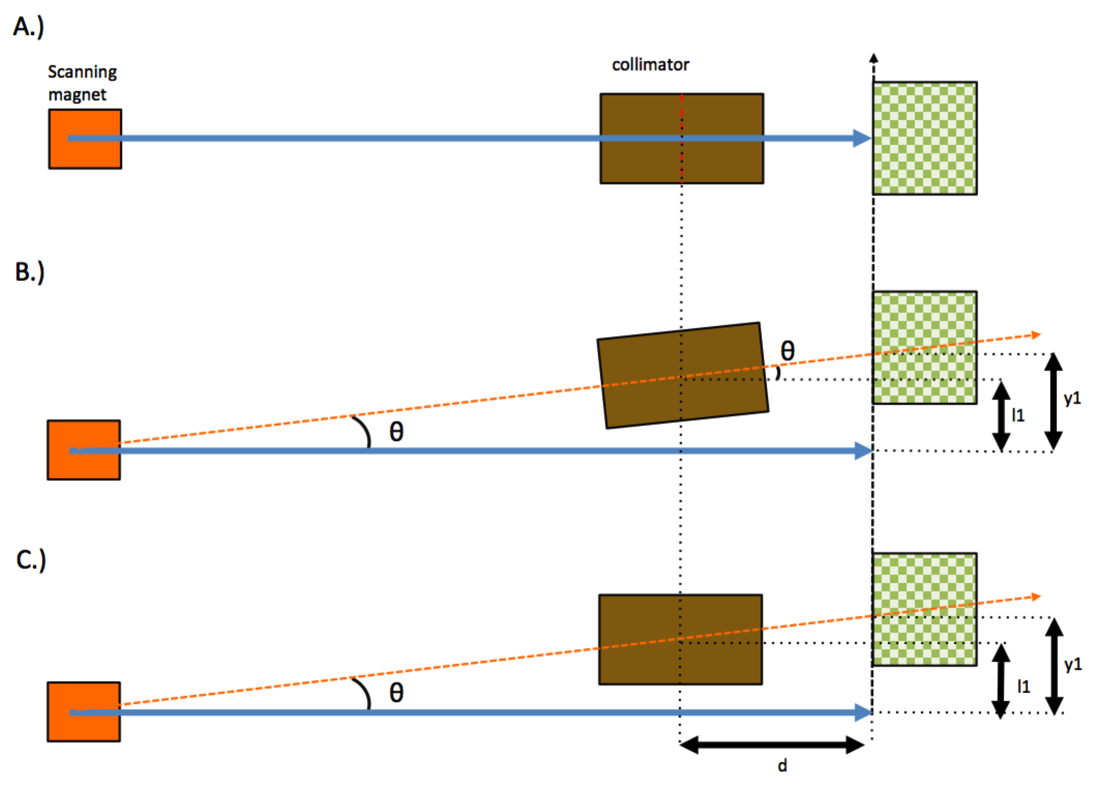


Figure 2‑4. A.) The beam spot when no magnetic field is applied at the scanning magnet. When a magnetic field is applied to the scanning magnet, the deflection angle is formed: B) the collimator is both repositioned and rotated to follow the beam deflection angle, C) the collimator is only repositioned.

The effect in the dose distributions of following the beam deflection angle is demonstrated in Figure 2‑5 and Figure 2‑6 for 100 and 150 MeV protons respectively.


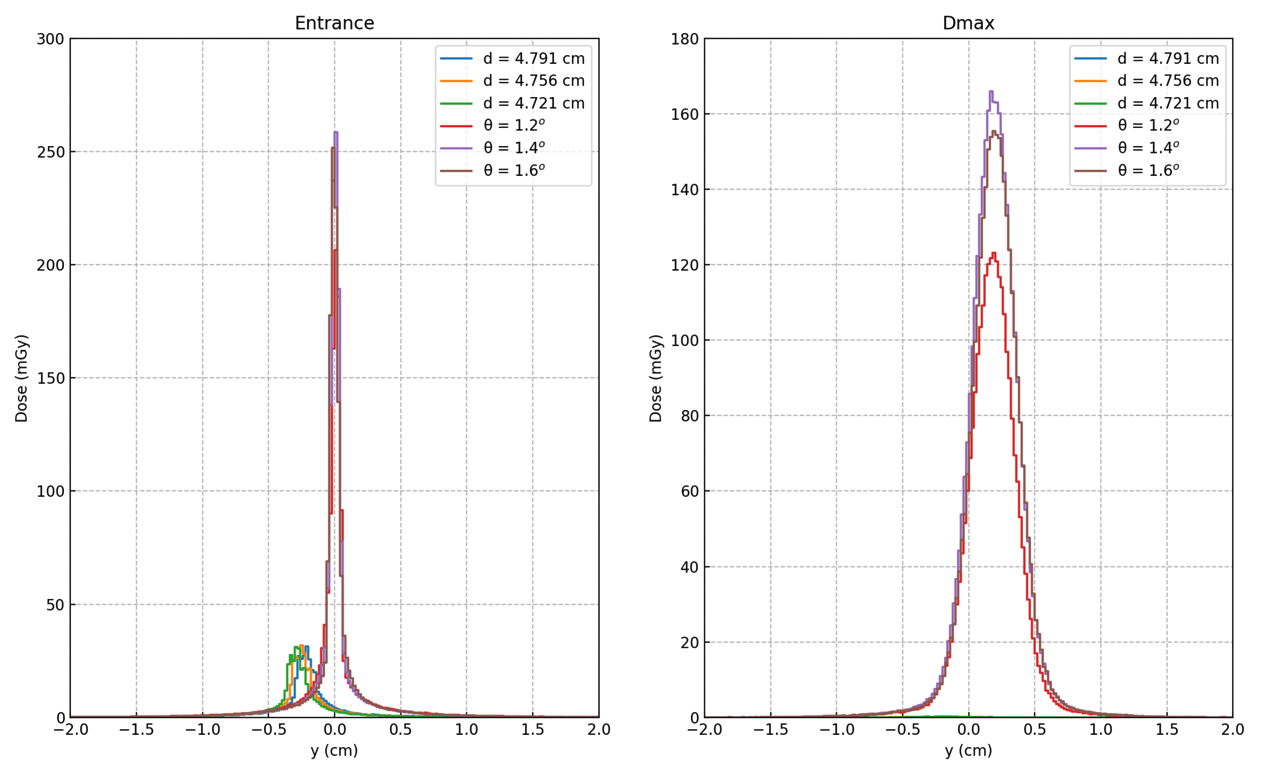


Figure 2‑5. Width-wise profiles of the dose distribution at the entrance of the phantom and at the D_max_ for a 100 MeV proton beam.


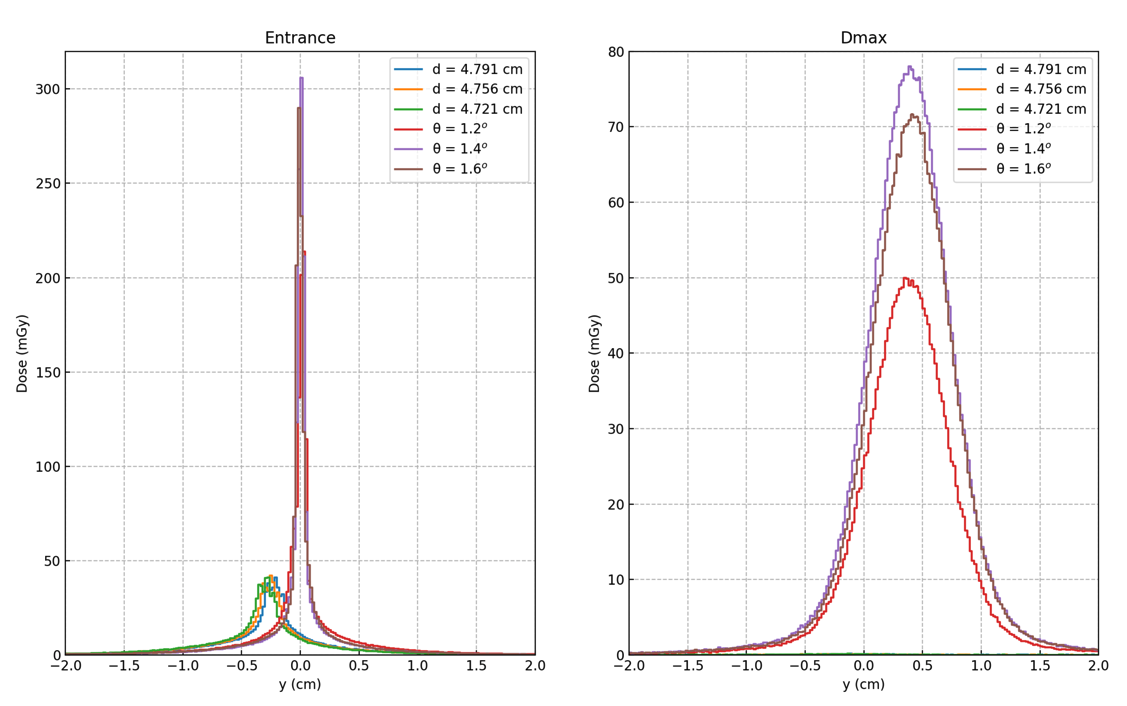


Figure 2‑6. Width-wise profiles of the dose distribution at the entrance of the phantom and at the D_max_ for a 150 MeV proton beam

## Comparing different collimator designs

The resulting minibeam from a parallel plate collimator, a two-plane collimator (see section 2.1.1 –in the main manuscript) is compared with the single slit collimator. Of particular interest is the two-plane collimator, where the order and the distance between the two stages are studied. The aperture collimator is not investigated as is considered to be equivalent to the single slit collimator. The slit width is 400 μm with a 5 cm slit length. Again, a voxelized water phantom at the isocentre with a voxel size of 0.5 x 0.1 x 1 mm^3^ is used for dose scoring purposed. The irradiation field consists of a line of 24 proton spots separated 3 mm at the isocentre; 500M protons are simulated per spot.

The length- and width-wise profiles for the different collimator designs are presented in Figure 2‑7 and Figure 2‑8 for the 100 and 150 MeV proton beams respectively.


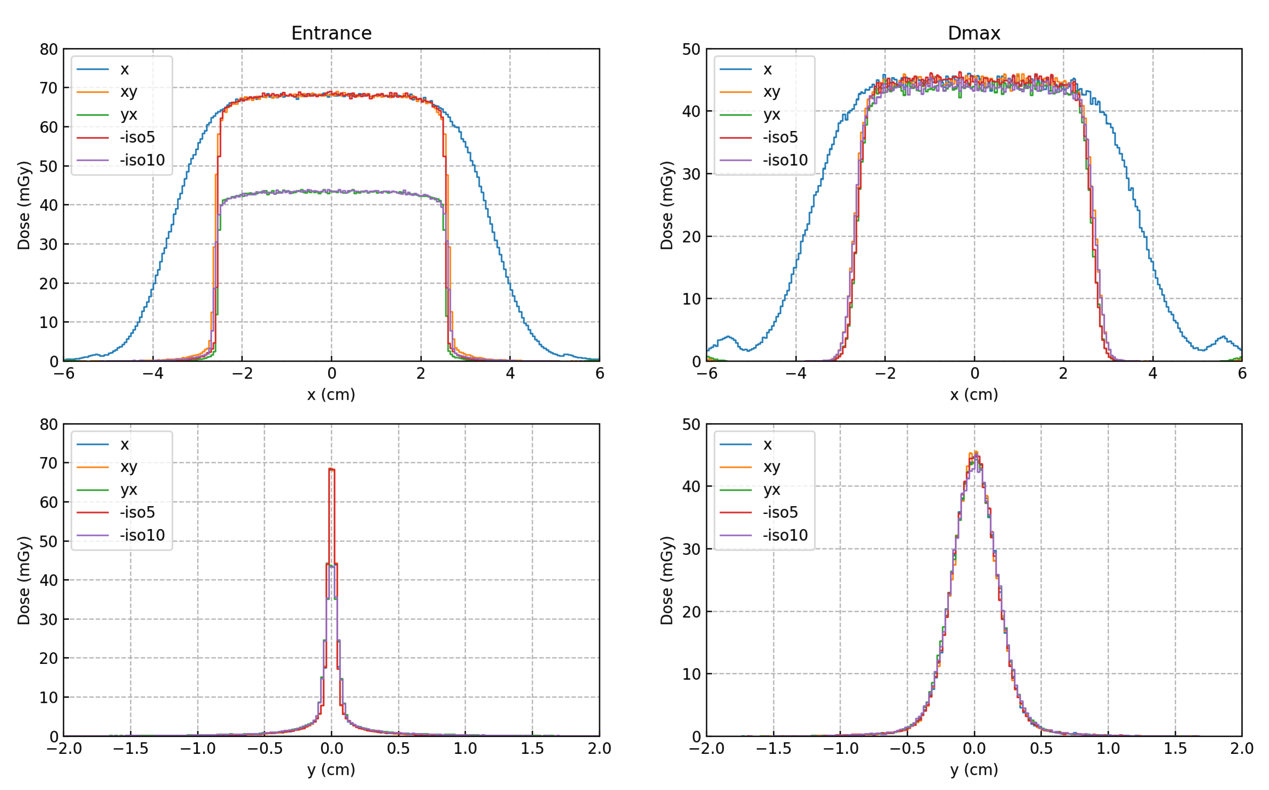


Figure 2‑7. Profiles of the dose distribution for the different collimator designs: single parallel collimator (‘x’), two stage collimator with the width (‘xy’) or length (‘yx’) collimator close to the phantom surface, and a single slit collimator at 5 cm of 10 cm from the phantom surface; for a proton beam of 100 MeV.


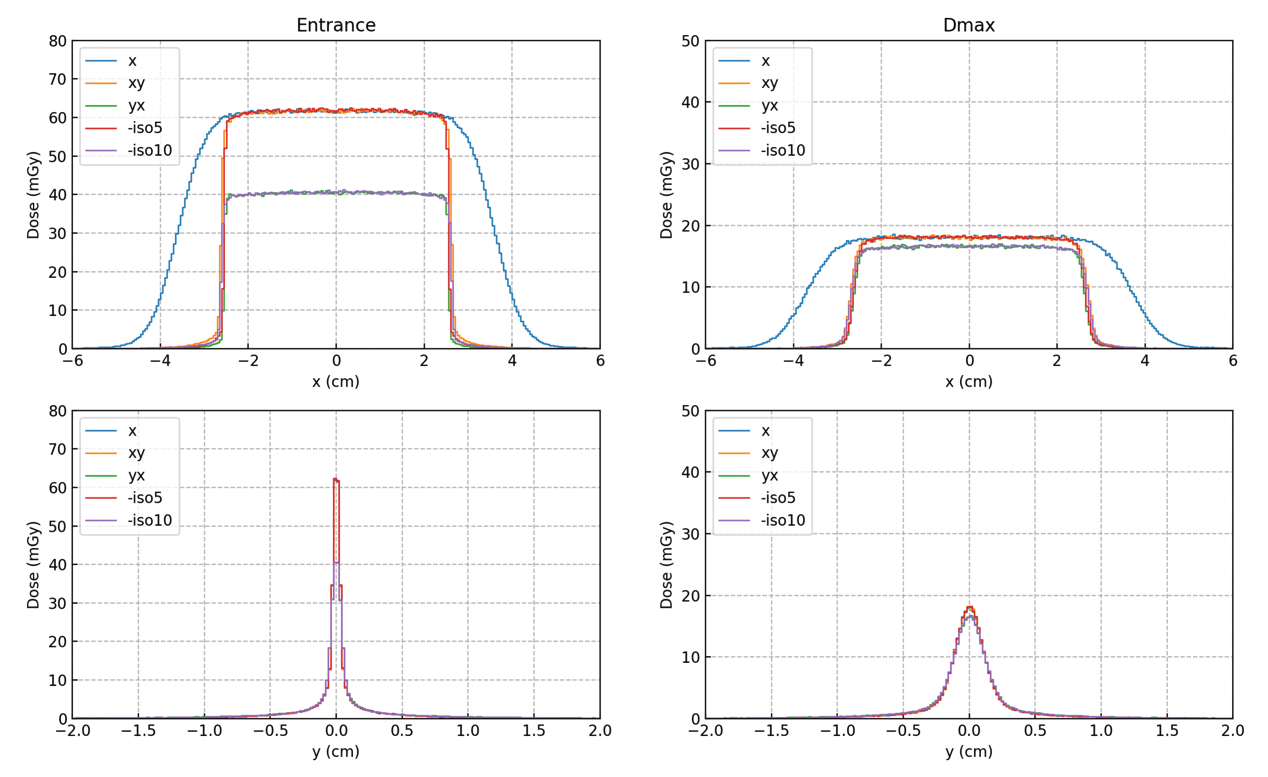


Figure 2‑8. Profiles of the dose distribution for the different collimator designs: single parallel collimator (‘x’), two stage collimator with the width (‘xy’) or length (‘yx’) collimator close to the phantom surface, and a single slit collimator at 5 cm of 10 cm from the phantom surface; for a proton beam of 150 MeV.

# Beam deflection angle

When the beam spots scanned using the scanning magnets, an angle is formed between the beam trajectory and the trajectory when no magnetic field is applied to the magnets. In many cases this beam deflection angle is required. It is therefore necessary to create a formula that allows to calculate the deflection angle as a function of the beam position for every spot. This could be calculated theoretically, but since not all the parameters required are known with the necessary accuracy, it is preferred to use the beam line model for this estimation.

Two thin scoring volumes are placed 1 m apart. By fitting a Gaussian on the screen we get the horizontal position of the beam. Then we can calculate the deflection of the beam using simple trigonometry.

The TOPAS simulation is comprised of the full ICPO beam line geometry and a proton beam of 100 MeV at the isocentre. Two scoring ‘screens’ (8x8x1 cm each screen, 800x800x1 voxels) counting the number of particles passing through are placed; one at the isocentre (Z_0_ = 0) and one at Z_0_ = 1 m (see Figure 3‑1). The screen is repositioned for every deflection angle so that the spot is at about the centre of the screen. This allows to have the same screen size for all deflection angles.


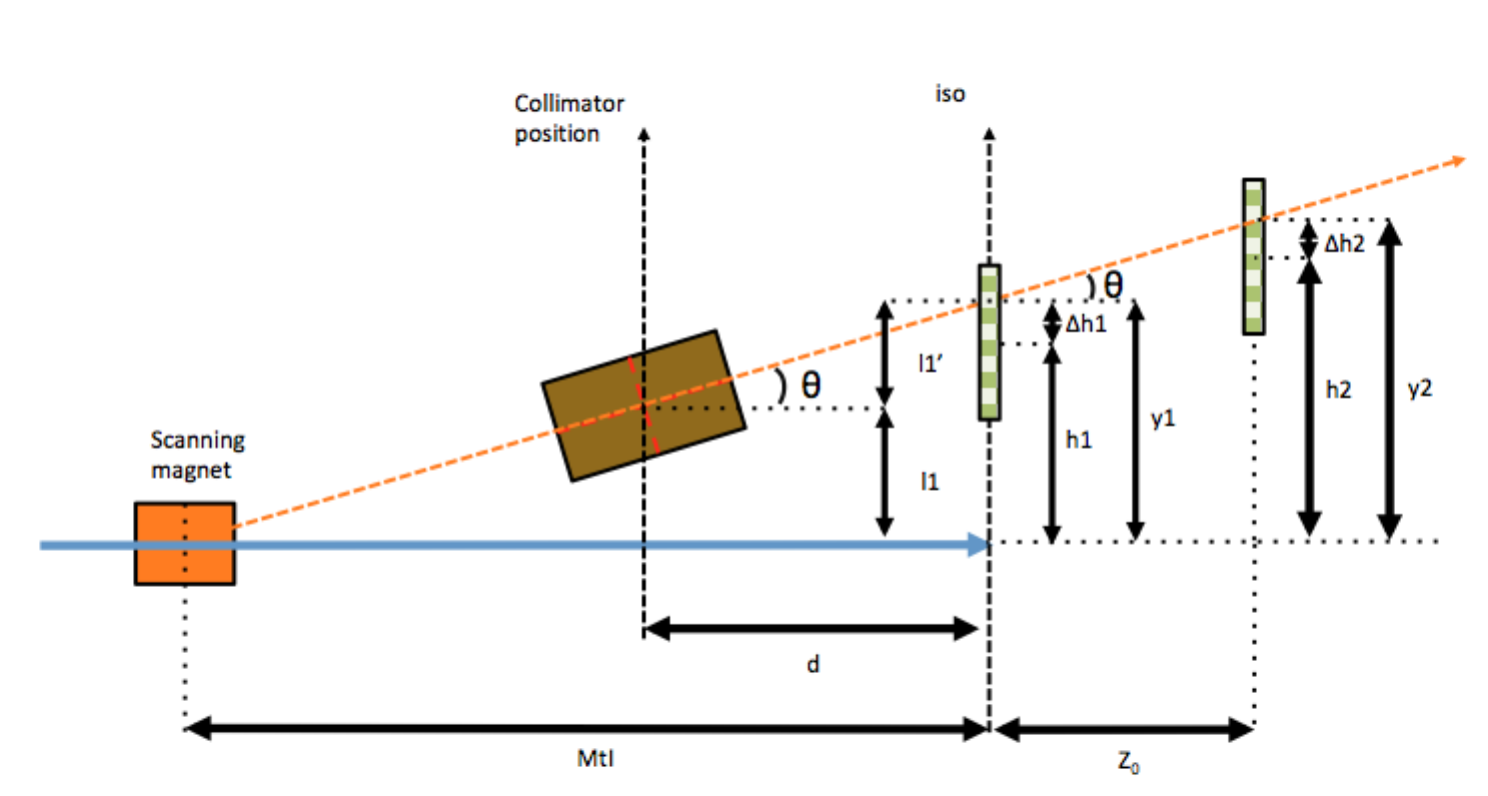


Figure 3‑1. Illustration of the geometry used for estimating the deflection angle. The screens are illustrated with the white-green checkers colour. Screen one is on the left and screen 2 on the right.

From Figure 3‑1, the deflection angle can be calculated as:

|  | $tan\theta=\frac{y_{2}-y_{1}}{Z_{0}}=\frac{\left( h_{2}+\Delta h_{2} \right)-\left( h_{1}+\Delta h_{1} \right)}{Z_{0}}$ | Eq. 2 |
| --- | --- | --- |

where $y_{1}$ and $y_{2}$ are the position of the beam on the screen at the isocentre and 1 m away, respectively; $h_{1}$ and $h_{2}$ is the offset of the screen at the isocentre and 1 m away, respectively; $Z_{0}$ is the distance between the two screens.

## Scanning Magnet 1 (SM1) i.e. scanning along x-axis

### E = 100 MeV

The screen position is changed so that for each deflection angle and related position, the spot is placed more or less at the centre of the screens. The spots on the screens can be seen at Figure 3‑2, with the Gaussian fit shown in Figure 3‑3.


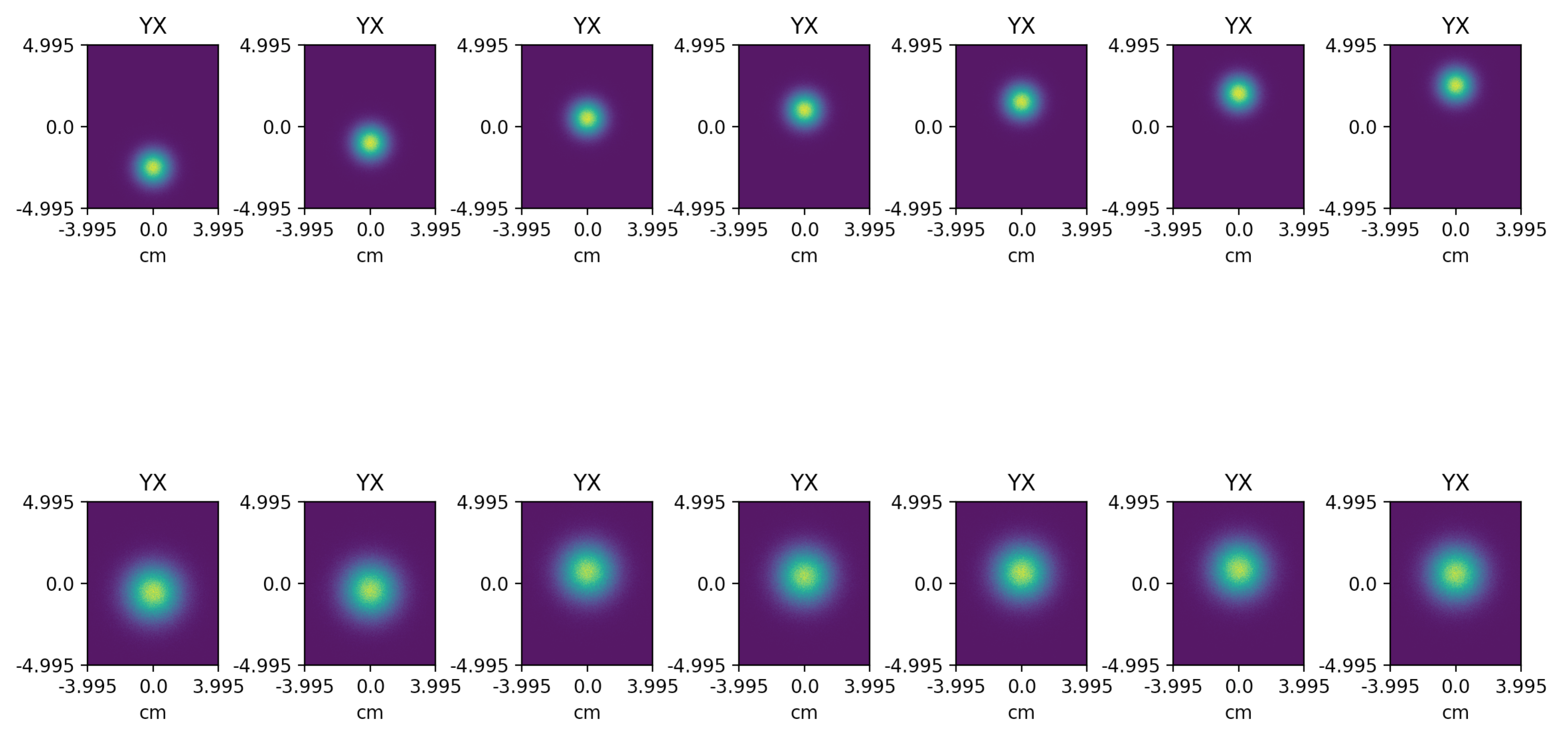


Figure 3‑2. Images of the beam passing the screens, (top) at isocentre and (bottom) 1 m away from the isocentre.


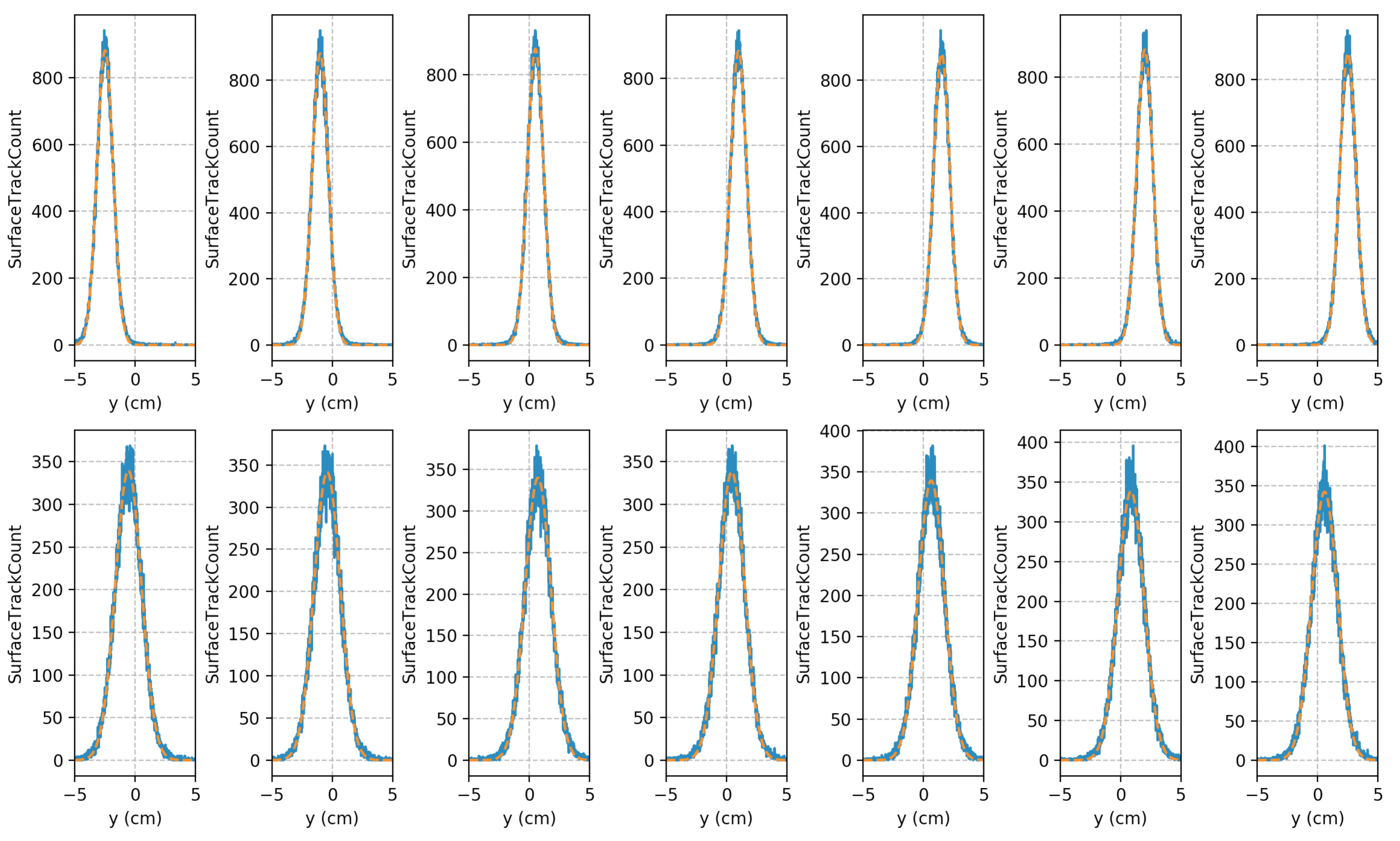

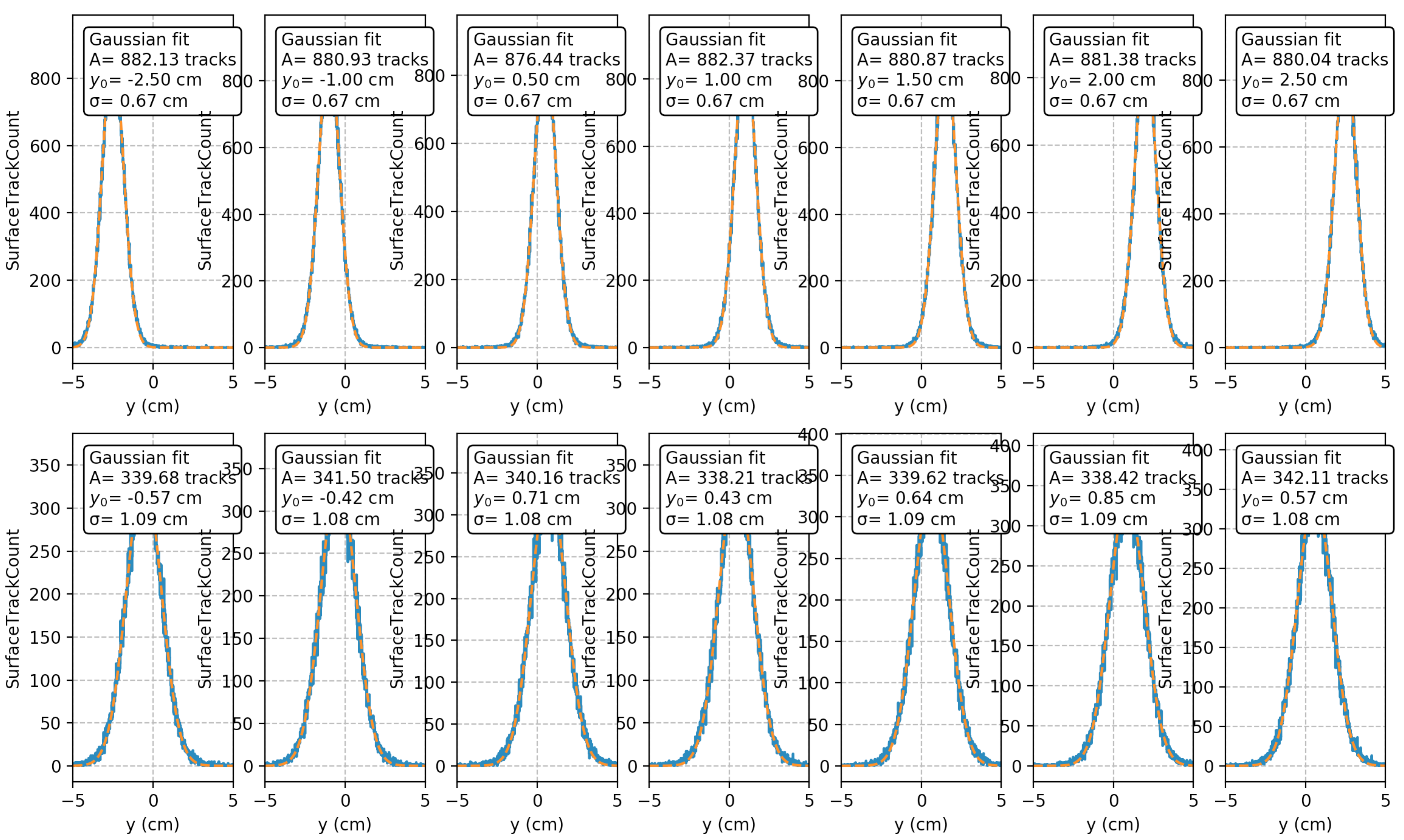


Figure 3‑3. Fitting Gaussians on the vertical profiles to estimate the beam position. TOP: data. BOTTOM: fit results.

By changing the spot position, we can get the calculated angle by using the formula in Eq. 2, filling Table 3.1.

Table 3‑1. Table with the data and results from the beam deflection angle calculations. First column is the spot position at the isocentre and the last column is the calculated deflection angle.

| $\mathbf{x}_{\mathbf{nominal}}\mathbf{(cm)}$ | $\boldsymbol{\Delta}\mathbf{h}_{\mathbf{1}}\mathbf{(cm)}$ | $\boldsymbol{\Delta}\mathbf{h}_{\mathbf{2}} \mathbf{(cm)}$ | $\mathbf{h}_{\boldsymbol{2}}$ (cm) | $\mathbf{x}_{\mathbf{2}} \left( \mathbf{cm} \right)$ | θ (deg) |
| --- | --- | --- | --- | --- | --- |
| -2.500 | -2.5025 | -0.5662 | -3.000 | -3.5662 | -0.60939 |
| -1.000 | -1.0020 | -0.4247 | -1.000 | -1.4247 | -0.24218 |
| 0.500 | 0.5007 | 0.7134 | 0.000 | 0.7134 | 0.12190 |
| 1.000 | 1.0014 | 0.4346 | 1.000 | 1.4346 | 0.24821 |
| 1.500 | 1.5012 | 0.6396 | 1.500 | 2.1396 | 0.36578 |
| 2.000 | 2.0020 | 0.8528 | 2.000 | 2.8528 | 0.48748 |
| 2.500 | 2.4992 | 0.5689 | 3.000 | 3.5689 | 0.61288 |

Finally the data are plotted and the best fit to the data is calculated (Figure 3‑4).


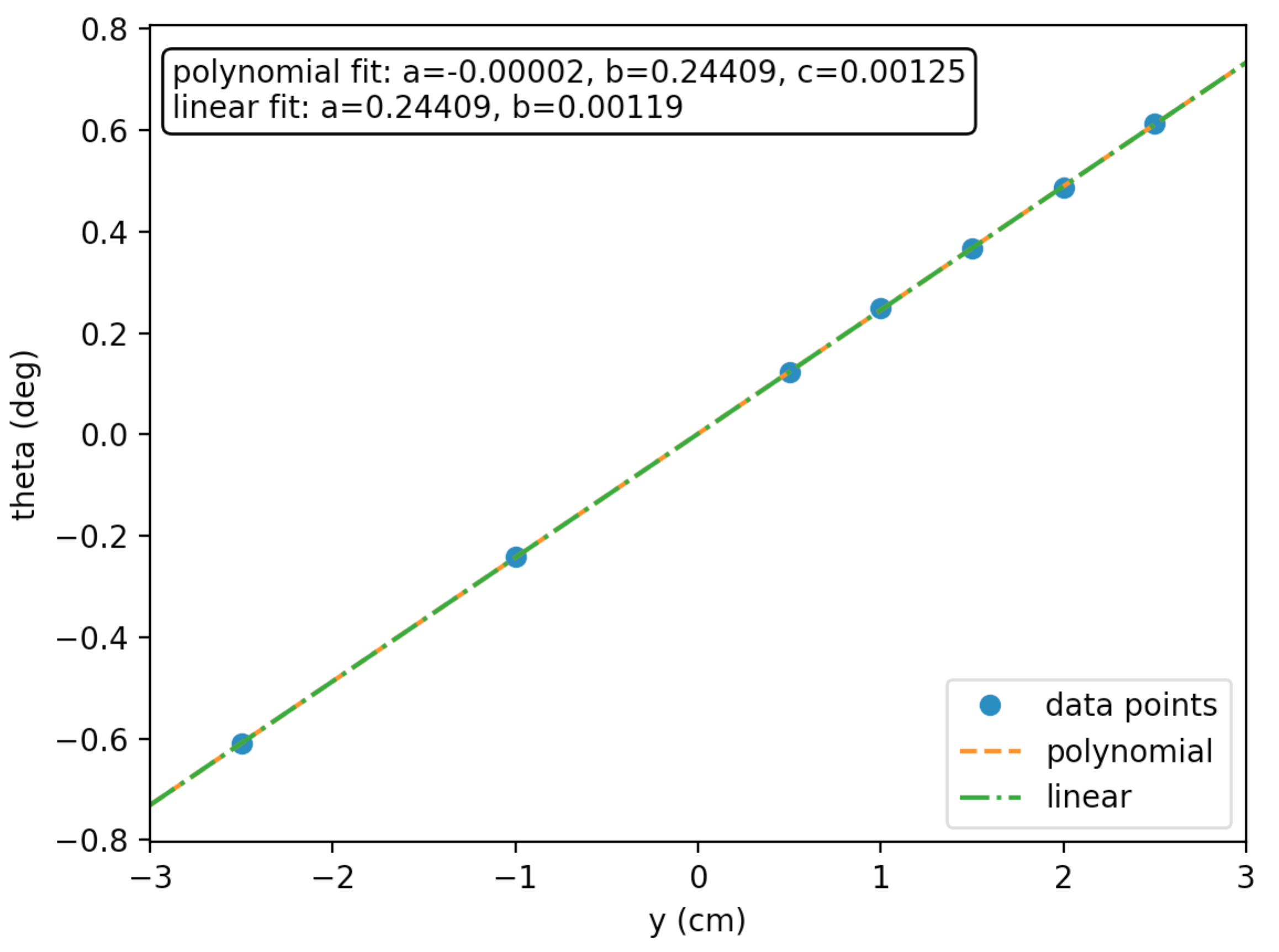


Figure 3‑4. Deflection angle as a function of spot position at the iso-centre. A polynomial and linear fit is attempted.

From Figure 3‑4 a linear relation between the spot position and the deflection angle is observed, as expected, due to the small angles involved.

|  | θ(x) = 0.24409 deg cm^-1^ x + 0.00119 deg | Eq. 3 |
| --- | --- | --- |

### E = 150 MeV

The method described in section 3.1.1 is also followed for a beam of 150 MeV.


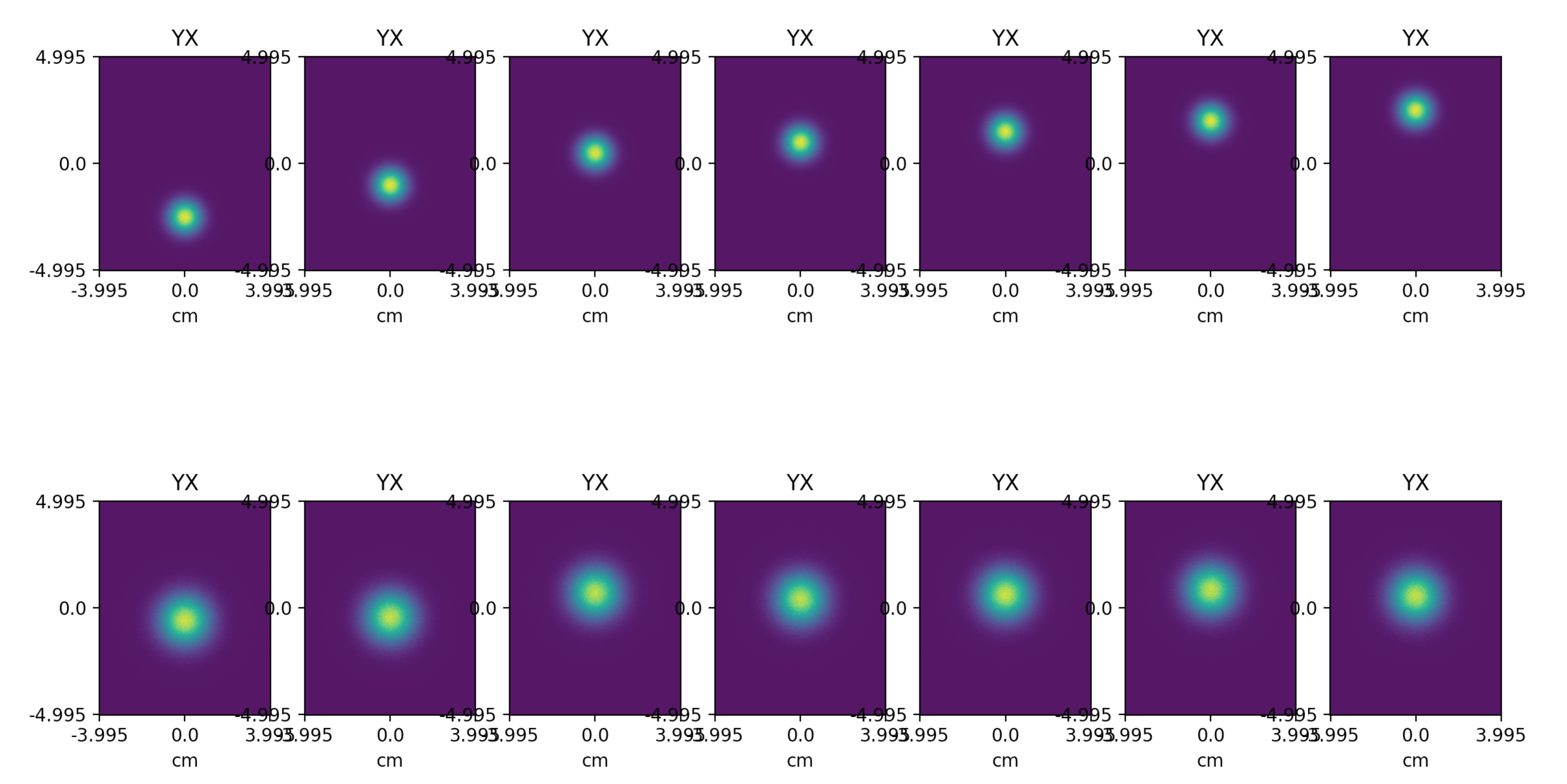


Figure 3‑5. Images of the beam passing the screens, (top) at isocentre and (bottom) 1 m away from the isocentre.


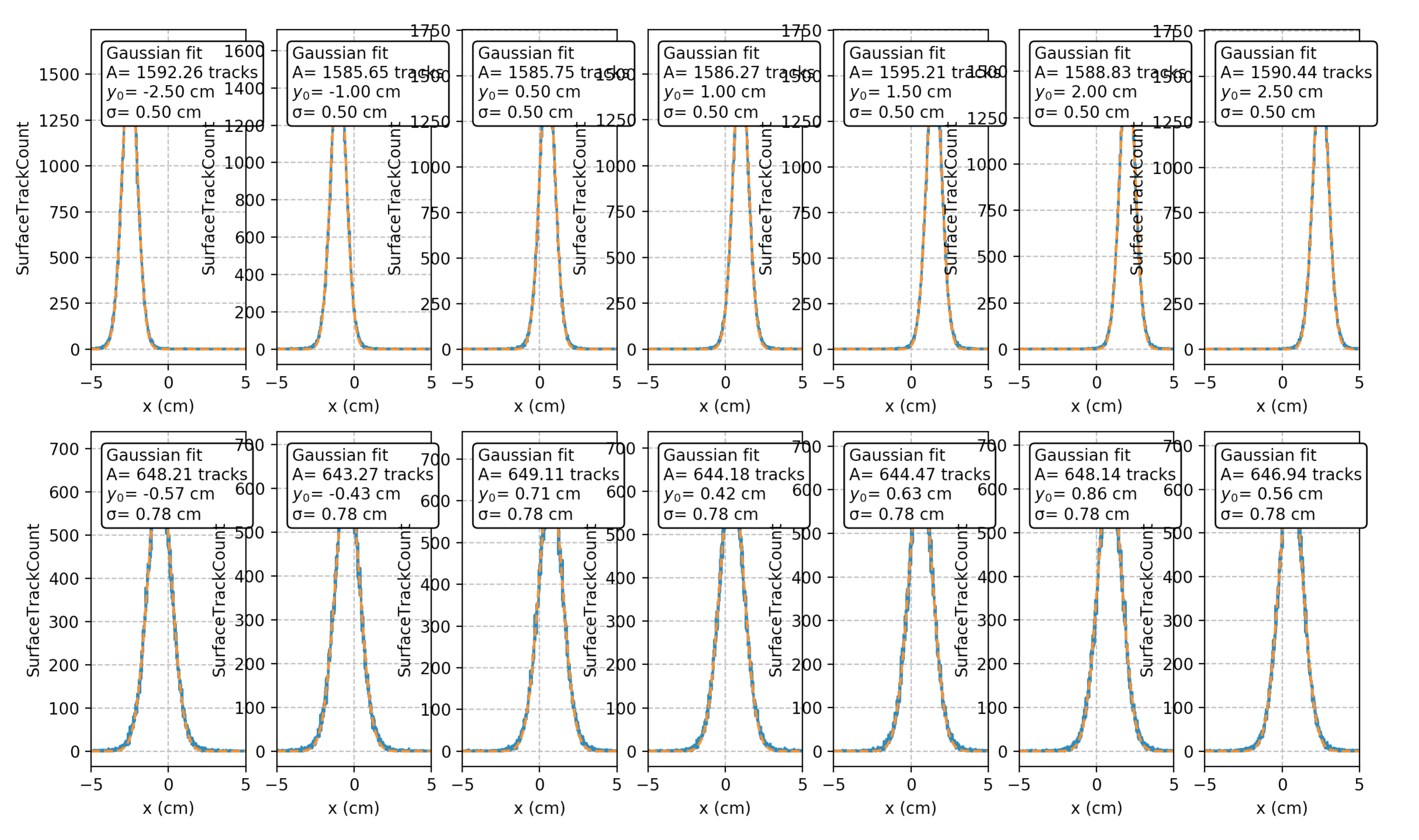


Figure 3‑6. Fitting Gaussians on the vertical profiles to estimate the beam position.

Table 3‑2. Table with the data and results from the beam deflection angle calculations. First column is the spot position at the isocentre and the last column is the calculated deflection angle.

| **x_nominal_ (cm)** | **Δh_1_ (cm)** | **Δh_2_ (cm)** | **h_2_ (cm)** | **x_2_ (cm)** | **θ (deg)** |
| --- | --- | --- | --- | --- | --- |
| -2.5 | -2.496348937 | -0.565465476 | -3 | -3.565465476 | -0.612535318 |
| -1 | -0.99965764 | -0.426455671 | -1 | -1.426455671 | -0.244535774 |
| 0.5 | 0.49994953 | 0.710555012 | 0 | 0.710555012 | 0.120667874 |
| 1 | 0.999135624 | 0.424612112 | 1 | 1.424612112 | 0.243778599 |
| 1.5 | 1.501713557 | 0.633855097 | 1.5 | 2.133855097 | 0.362185599 |
| 2 | 1.998430044 | 0.85674079 | 2 | 2.85674079 | 0.491763757 |
| 2.5 | 2.496826064 | 0.564126037 | 3 | 3.564126037 | 0.611494621 |


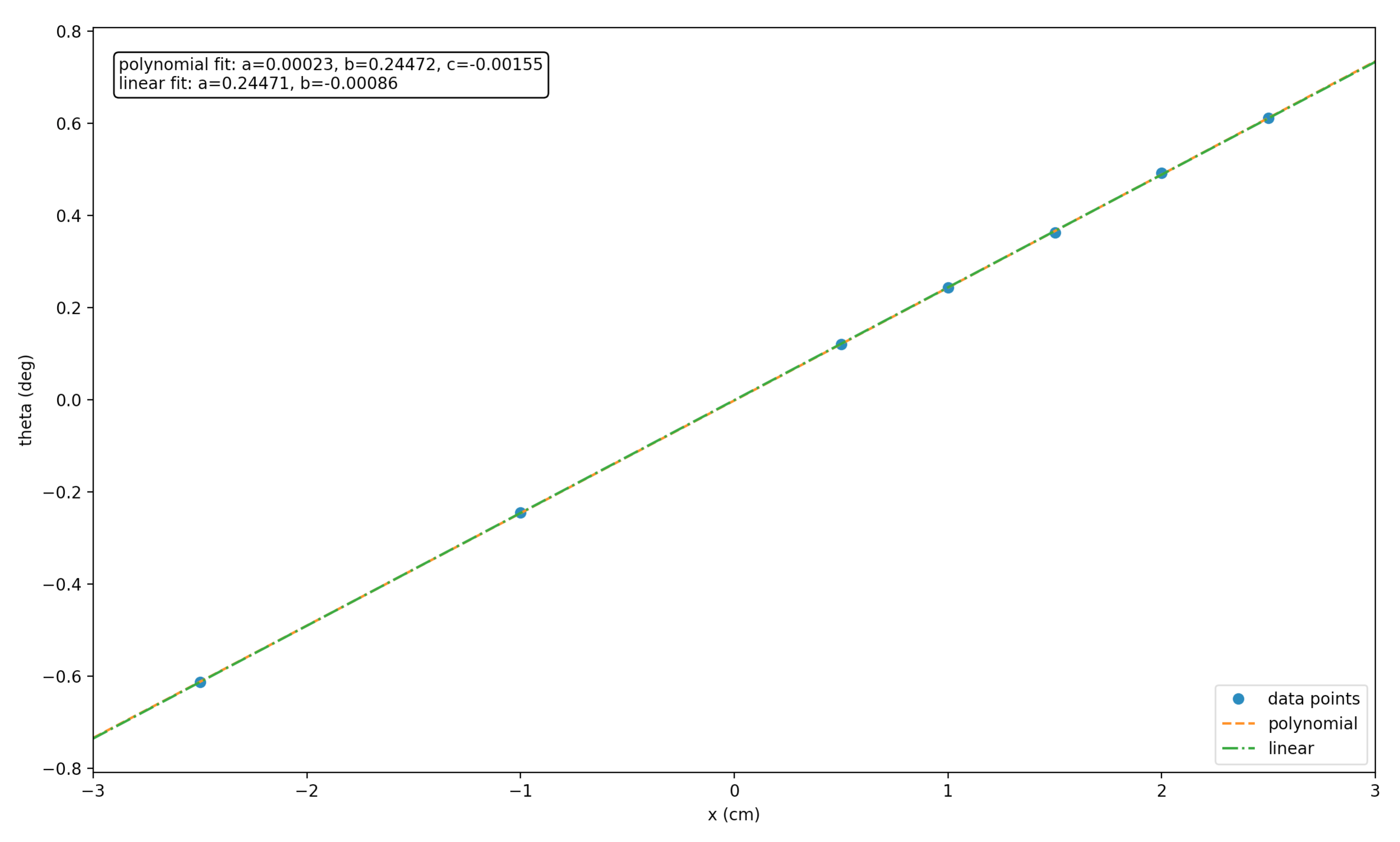


Figure 3‑7 Deflection angle as a function of spot position at the iso-centre, with a polynomial and linear fit.

Again, from Figure 3‑7 a linear relation between the spot position and the deflection angle is observed, as expected, due to the small angles involved.

|  | θ(x) = 0.24471 deg cm^-1^ x - 0.00086 deg | Eq. 4 |
| --- | --- | --- |

## Calculating the slit positions


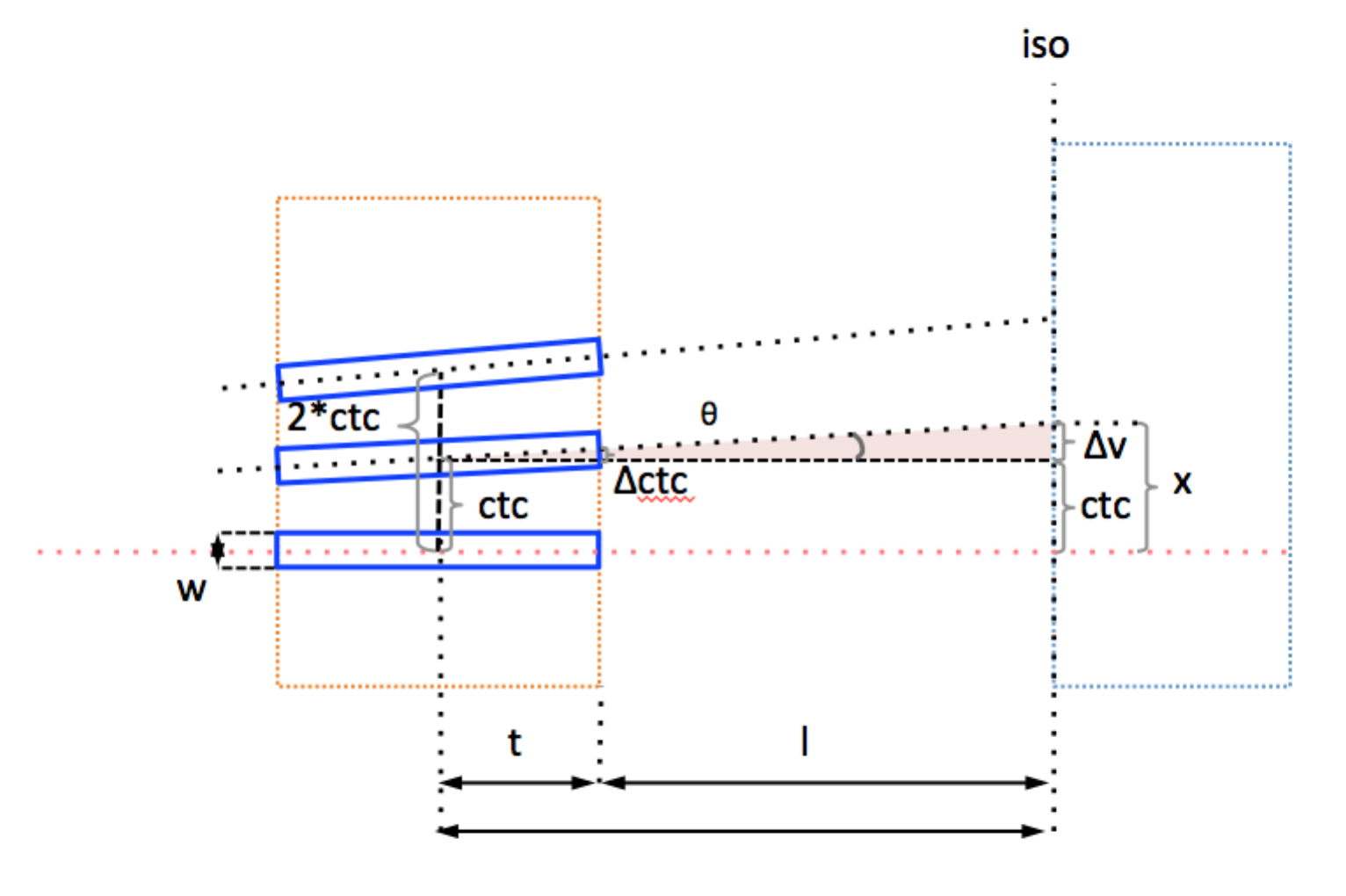

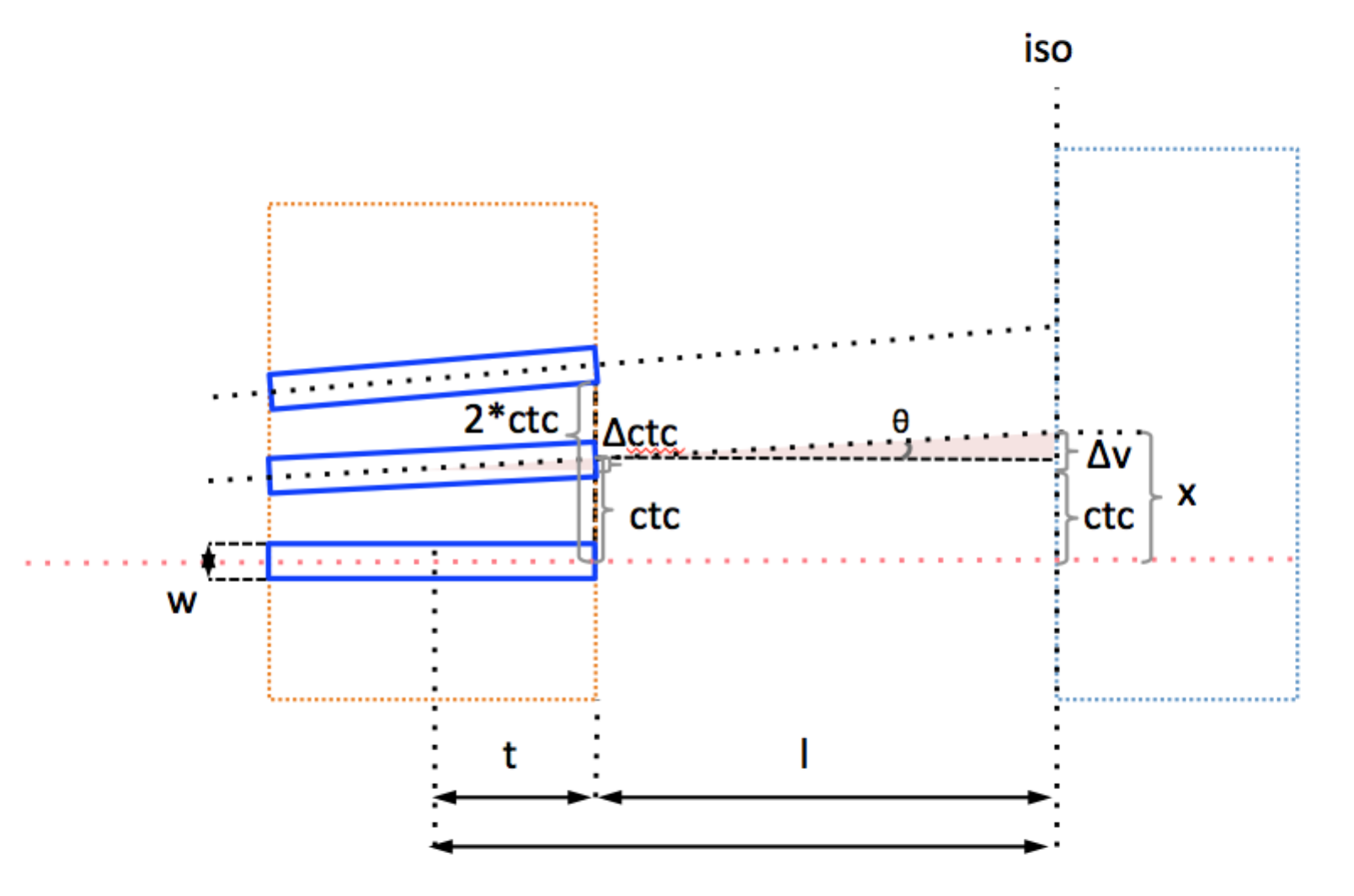


Figure 3‑8. Geometry of the multislit collimator for the calculation of the slits’ positions and angles.

We can calculate the angle of the slit in relation to the centre-to-centre distance of the slits and the distance from the isocentre (t+l). We will assume a linear relation between the deflection angle θ and the position on the isocentre, x:

$$\theta^{\deg}\left( x \right)=\alpha\cdot x+b \Longrightarrow$$

$$x\left( \theta\right)=\frac{\theta^{\deg}-b}{\alpha}$$

Also from the pink triangle we get

$$tan\theta=\frac{\Delta v}{l+t}\Longrightarrow$$

$$\Delta v=(l+t)\cdot tan\theta$$

and finally

$$x\left( \theta\right)=\Delta v+ctc$$

Combining the second and the last relation we have

$$\Delta v+ctc=\frac{\theta^{\deg}-b}{\alpha}\Longrightarrow$$

$$(l+t)\cdot tan\theta+ctc=\frac{\theta^{\deg}-b}{\alpha}\Longrightarrow$$

And having the small angle approximation for the tangent (tanθ ≈ πθ/180, where θ is in degrees)

$$(l+t)\cdot\frac{\pi}{180}\theta^{\deg}+ctc=\frac{\theta^{\deg}-b}{\alpha}\Longrightarrow$$

$$\alpha\cdot(l+t)\cdot\frac{\pi}{180}\theta^{\deg}+\alpha\cdot ctc=\theta^{\deg}-b\Longrightarrow$$

$$\alpha\cdot ctc+b=\theta^{\deg}\cdot\left( 1-\alpha\cdot(l+t)\cdot\frac{\pi}{180} \right)\Longrightarrow$$

$$\theta^{\deg}=\frac{\alpha\cdot ctc+b}{1-\alpha\cdot(l+t)\cdot\frac{\pi}{180}}\Longrightarrow$$

That gives the angle as a function of the ctc and the distance from the isocentre [l+t]. Also, the position of the front point of the collimator will be

$$x_{\mathrm{front}}=ctc+\Delta ctc=ctc+t\cdot\tan\theta$$

Sometimes the ctc and distance to the isocentre has to be measured from the front face of the collimator (see Figure 3‑5 bottom). In this case the angle is

$$\theta^{\deg}=\frac{\alpha\cdot ctc+b}{1-\alpha\cdot l\cdot\frac{\pi}{180}}$$

and the position of the centre of the collimator

$$x_{centre}=ctc-\Delta ctc=ctc-t\cdot\tan\theta$$

Note that the angle calculated in the two cases is not exactly the same, as the position of the collimator slightly changes.
